# Global fine-scale changes in ambient NO_2_ during COVID-19 lockdowns

**DOI:** 10.1038/s41586-021-04229-0

**Published:** 2022-01-19

**Authors:** Matthew J. Cooper, Randall V. Martin, Melanie S. Hammer, Pieternel F. Levelt, Pepijn Veefkind, Lok N. Lamsal, Nickolay A. Krotkov, Jeffrey R. Brook, Chris A. McLinden

**Affiliations:** 1 Department of Physics and Atmospheric Science, Dalhousie University, Halifax, Nova Scotia Canada; 2 Department of Energy, Environmental & Chemical Engineering, Washington University in St. Louis, St. Louis, MO USA; 3 Harvard-Smithsonian Center for Astrophysics, Cambridge, MA USA; 4 Royal Netherlands Meteorological Institute (KNMI), De Bilt, Netherlands; 5 University of Technology Delft, Delft, Netherlands; 6 National Center for Atmospheric Research, Boulder, CO USA; 7 Department of Geoscience and Remote Sensing, Delft University of Technology, Delft, Netherlands; 8 NASA Goddard Space Flight Center, Greenbelt, MD USA; 9 Universities Space Research Association, Columbia, MD USA; 10 Dalla Lana School of Public Health, University of Toronto, Toronto, Ontario Canada; 11 Department of Chemical Engineering and Applied Chemistry, University of Toronto, Toronto, Ontario Canada; 12 Environment and Climate Change Canada, Toronto, Ontario Canada

**Keywords:** Atmospheric chemistry, Environmental monitoring

## Abstract

Nitrogen dioxide (NO_2_) is an important contributor to air pollution and can adversely affect human health^1–9^. A decrease in NO_2_ concentrations has been reported as a result of lockdown measures to reduce the spread of COVID-19^10–20^. Questions remain, however, regarding the relationship of satellite-derived atmospheric column NO_2_ data with health-relevant ambient ground-level concentrations, and the representativeness of limited ground-based monitoring data for global assessment. Here we derive spatially resolved, global ground-level NO_2_ concentrations from NO_2_ column densities observed by the TROPOMI satellite instrument at sufficiently fine resolution (approximately one kilometre) to allow assessment of individual cities during COVID-19 lockdowns in 2020 compared to 2019. We apply these estimates to quantify NO_2_ changes in more than 200 cities, including 65 cities without available ground monitoring, largely in lower-income regions. Mean country-level population-weighted NO_2_ concentrations are 29% ± 3% lower in countries with strict lockdown conditions than in those without. Relative to long-term trends, NO_2_ decreases during COVID-19 lockdowns exceed recent Ozone Monitoring Instrument (OMI)-derived year-to-year decreases from emission controls, comparable to 15 ± 4 years of reductions globally. Our case studies indicate that the sensitivity of NO_2_ to lockdowns varies by country and emissions sector, demonstrating the critical need for spatially resolved observational information provided by these satellite-derived surface concentration estimates.

## Abstract (web summary)

The satellite instrument TROPOMI is used to assess ambient NO_2_ levels at approximately one-kilometre resolution across 215 cities worldwide during COVID-19 lockdowns, finding about 30% lower NO_2_ concentrations in countries with strict lockdowns.

## Main

Nitrogen dioxide (NO_2_) is an important contributor to air pollution as a primary pollutant and as a precursor to ozone and fine particulate matter production. Human exposure to elevated NO_2_ concentrations is associated with a range of adverse outcomes such as respiratory infections^2–4^, increases in asthma incidence^5,6^, lung cancer^7^ and overall mortality^8,9^. NO_2_ observations indicate air quality relationships with combustion sources of pollution such as transportation^6,21^. Initial investigations found substantial decreases in the atmospheric NO_2_ column from satellite observations^10–16^ and in ambient NO_2_ concentrations from ground-based monitoring^17–20^ during lockdowns enacted to reduce the spread of COVID-19. However, questions remain about the relationship of atmospheric columns with health- and policy-relevant ambient ground-level concentrations, and about the representativeness of sparse ground-based monitoring for broad assessment. Thus, there is need to relate satellite observations of NO_2_ columns to ground-level concentrations. It is also important to consider the effect of meteorology on recent NO_2_ changes^22^ and to quantify NO_2_ changes due to COVID-19 interventions in the context of longer-term trends^23^. Furthermore, air quality monitoring sites tend to be preferentially located in higher-income regions, raising questions about how NO_2_ changed in lower-income regions where larger numbers of potentially susceptible people reside. Estimates of changes in ground-level NO_2_ concentrations derived from satellite remote sensing would fill gaps between ground-based monitors, offer valuable information in regions with sparse monitoring, and more clearly connect satellite observations with ground-level ambient air quality.

Previous satellite-derived estimates of ground-level NO_2_ used information on the vertical distribution of NO_2_ from a chemical transport model to relate satellite NO_2_ column densities to ground-level concentrations^24–26^. Recent work improved upon this technique by allowing the satellite column densities to constrain the vertical profile shape, allowing for more accurate representation of sub-model-grid variability, reducing the sensitivity to model resolution and simulated profile shape errors, and improving agreement between the satellite-derived ground-level concentrations and in situ monitoring data^27^. Applying this technique to examine changes in NO_2_ during lockdowns bridges the gap between previous studies focusing on either ground monitors or satellite column densities, thus providing a more complete and reliable picture of the changes in exposure.

Since 2005, the gold standard for satellite NO_2_ observations has been the Ozone Monitoring Instrument (OMI) on board NASA’s Earth Observing System Aura satellite^28,29^. The newest remote sensing spectrometer, the European Space Agency’s TROPOspheric Monitoring Instrument (TROPOMI)^30^ on the Copernicus Sentinel 5p satellite, has been providing NO_2_ observations with finer spatial resolution and higher instrument sensitivity since 2018. These attributes allow the generation of TROPOMI NO_2_ maps at 100 times finer resolution (approximately 1 × 1 km^2^) with a one-month averaging period^31,32^, an improvement over the spatial and temporal averaging needed for accurate OMI maps (typically approximately 10 × 10 km^2^ over one year)^24^. Concurrently, the excellent stability of the OMI instrument over the last 15 years provides an ideal dataset for long-term trend analysis^28,33^ that offers context for recent TROPOMI data.

Lockdown restrictions act as an experiment about the efficacy of activity reductions on mitigating air pollution. The Oxford COVID-19 Government Response Tracker (OxCGRT, https://www.bsg.ox.ac.uk/research/research-projects/coronavirus-government-response-tracker#data) has been monitoring government-imposed restrictions, and studies have indicated that NO_2_ decreases were larger for cities in countries with strict lockdowns^34^. However, there is limited information on lockdown stringency on sub-national levels or on how various emission sectors respond to lockdowns. An observation-based metric for lockdown intensity could provide useful information for examining lockdowns on city-level scales or for examining the effects on air quality that are associated with lockdowns in different emission sectors.

Here we leverage the high spatial resolution of TROPOMI to infer global ground-level NO_2_ estimates at, to our knowledge, an unprecedented spatial resolution sufficient to assess individual cities worldwide, and to examine changes in ground-level NO_2_ occurring during COVID-19 lockdowns from January–June 2020. Case studies presented here demonstrate how the satellite-based estimates provide information on important spatial variability in lockdown-driven NO_2_ changes, and in the NO_2_ response to lockdowns in various emissions sectors. We also use TROPOMI to provide fine-scale structure to the long-term record of OMI observations (2005–2019), which provides an opportunity to examine trends in ground-level NO_2_ over the last 15 years to provide context for the recent changes.

## Global NO_2_ concentrations and trends

**Fig. 1:**
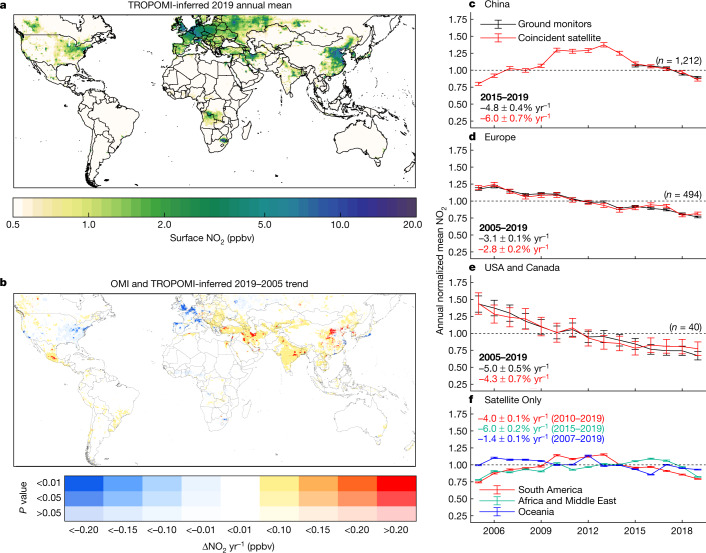
Satellite-derived ground-level NO_2_ concentrations. **a**, TROPOMI-derived 2019 annual mean ground-level NO_2_ concentrations at approximately 1 × 1 km^2^ resolution. **b**, Trend in OMI and TROPOMI-derived annual mean ground-level concentrations from 2005–2019. The colour intensity represents the statistical significance of the trend. **c**–**e**, Population-weighted mean NO_2_ from ground monitors and from satellite-derived NO_2_ sampled at ground-monitor locations in China (**c**), Europe (**d**) and North America (**e**), normalized by the mean concentration during the period where ground-monitor data are available. The black (ground-derived) and red (satellite-derived) values give the trends for the period where ground-monitor data are available. Only monitors with data available over the entire time period are included. Error bars represent population-weighted standard deviations. **f**, Population-weighted mean satellite-inferred ground-level NO_2_ concentrations in South America, Africa and the Middle East, and Oceania. Trends during the given time periods are given at top. Time periods were chosen to reflect the most recent years where a consistent trend is observed. Error bars represent uncertainties in population-weighted means using a bootstrapping method.

Global annual mean TROPOMI-derived ground-level NO_2_ concentrations for 2019 provide an initial baseline (Fig. 1). The excellent resolution (~1 × 1 km^2^) of ground-level NO_2_ concentrations reveal pronounced heterogeneity (Supplementary Figs. 1–7). NO_2_ enhancements are apparent over urban and industrial regions. TROPOMI-derived ground-level concentrations exhibit consistency with in situ observations (*r* = 0.71, *N* = 3,977, in situ versus satellite slope = 0.97 ± 0.02), as shown in Supplementary Fig. 8. Neglecting the spatial and temporal variability in the NO_2_ column-to-surface relationship degrades the consistency with ground monitors (slope = 0.78 ± 0.01), demonstrating the importance of relating satellite columns to surface concentrations for exposure assessment.

Examination of long-term changes in air pollution offers context for changes during COVID-19 lockdowns (Fig 1, Supplementary Figs. 1–7). Satellite-derived NO_2_ concentrations decreased from 2005–2019 in urban areas across most of the USA and Europe, eastern China, Japan, and near Johannesburg, South Africa, largely reflecting emission controls on vehicles and power generation. NO_2_ increases are observed in Mexico, the Alberta oil sands region in northern Canada, and throughout the Balkan peninsula, central and northern China, India and the Middle East, broadly consistent with reported trends in ground-monitor data^35–37^. Trends in China can be separated into three regimes: ground-level concentrations increased in China from 2005–2010, plateaued from 2010–2013, and decreased from 2013–2019. This change was driven by stricter vehicle and power generation emission standards^38^ and is consistent with observed changes in NO_2_ columns^39,40^. Similarly, concentrations increased in urban and industrial areas of South America from 2005–2010, and in South Africa and the Middle East from 2005–2015, and decreased in more recent years. Maps of trends in these regions for these time periods are shown in Supplementary Fig. 9. Concentrations in India increased across both time periods owing to increasing coal-powered electricity demands and growing industrial emissions^41^. Trends in population-weighted NO_2_ concentrations, used to represent population NO_2_ exposure, were calculated using ground monitors and coincidently sampled satellite observations in North America, Europe and China. Satellite-derived concentrations exhibit decreasing trends (−2.8 ± 0.2% yr^−1^ in Europe 2005–2019, −4.3 ± 0.7% yr^−1^ in North America 2005–2019, and −6.0 ± 0.7% yr^−1^ in China 2015–2019) that agree well with trends in the ground-monitor data (within 0.7% yr^−1^ in North America, 0.3% yr^−1^ in Europe, and 1.2% yr^−1^ in China).

## Regional NO_2_ changes during lockdowns

**Fig. 2:**
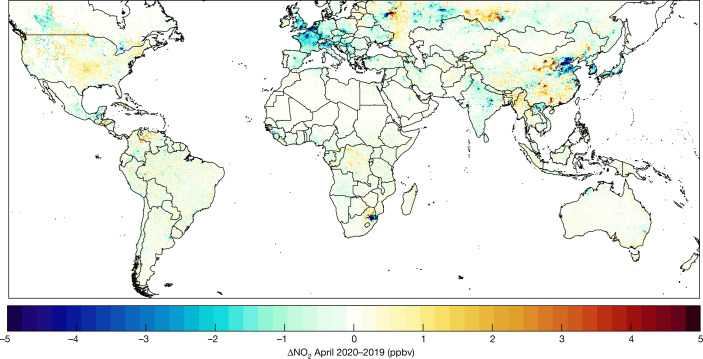
Differences in April mean ground-level NO_2_ from 2020 to 2019. Concentrations derived using TROPOMI observations gridded at approximately 1 × 1-km^2^ resolution.

**Fig. 3:**
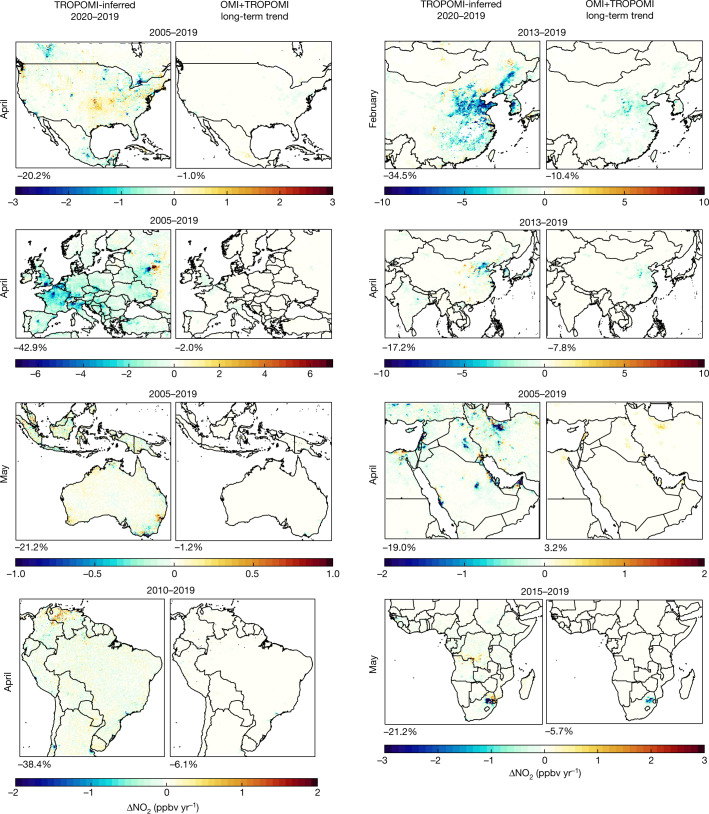
Changes in ground-level NO_2_ during lockdowns. Left in each pair of images, TROPOMI-derived monthly mean NO_2_ differences from 2020–2019 at approximately 1 × 1 km^2^. Right, OMI+TROPOMI-derived NO_2_ trends. Annual mean long-term trends are corrected for seasonal variation. The time periods for trend calculations in each region were chosen to reflect the most recent years where a consistent trend is observed and are indicated above the maps. Value under each panel represents population-weighted mean difference for the given region.

Figure 2 shows the April 2020 to April 2019 difference between mean ground-level NO_2_ concentrations derived from TROPOMI observations. NO_2_ concentrations are lower in most regions in 2020 than in 2019, particularly over urban areas, with global population-weighted mean concentrations decreasing by 16% in 2020 relative to 2019. Fig. 3 shows regional maps focusing on the month with the largest change in population-weighted regional mean concentration for each region, with an additional period included for China, as lockdown restrictions occurred earlier than in other countries. Regional population-weighted mean concentrations decreased by 17–43%. The largest decreases occur in China in February with concentration decreases exceeding 10 parts per billion by volume (ppbv) and substantial decreases persisting in eastern urban areas through April. Thus these lockdown measures temporarily bolstered the decreasing trends across North America^42^ and Europe^25^ over the last two decades and in China since 2012^43^, owing to technological advances in vehicles and power generation, while temporarily buffering changes from increasing energy demands in India and the Middle East^40,44,45^. NO_2_ increases in April 2020 in central China (Chengdu and Chongqing) because lockdowns began lifting during this time.

Figure 3 shows maps of long-term NO_2_ trends for context. In most regions, the observed changes during COVID-19 restrictions exceed the expected year-to-year differences observed in the long-term trends (Table 1). 2020–2019 population-weighted mean concentration changes are lower than long-term trends by factors of 17 ± 7 in North America, 19 ± 2 Europe, of 2.9 ± 0.6 in Africa and the Middle East, of 3.6 ± 0.6 in Asia, 8 ± 7 in South America, and 2 ± 2 in Oceania.

**Table 1:** TROPOMI-derived, population-weighted ground-level NO_2_ data

Country/region	Month with greatest 2020–2019 change	Monthly population-weighted mean NO_2_ concentration 2019 (ppbv)	Monthly population-weighted mean 2020–2019 difference (ppbv)	Expected 2020–2019 change from meteorology (ppbv)	Long-term trend in population-weighted NO_2_^a^ (ppbv/year)	Ratio of 2020–2019 difference to long-term trend (years)
China^b^	January	9.5 ± 0.3	−2.7 ± 0.3	0.057 ± 0.03	−0.8 ± 0.1	3.4 ± 0.6
India^b^	June	0.96 ± 0.06	−0.29 ± 0.03	−0.062 ± 0.002	0.017 ± 0.005	na
USA	March	3.0 ± 0.1	−0.40 ± 0.08	−0.12 ± 0.01	−0.119 ± 0.009	3.4 ± 0.7
Indonesia^b^	June	1.24 ± 0.04	−0.3 ± 0.3	−0.031 ± 0.007	−0.016 ± 0.006	20 ± 20
Brazil^c^	April	1.01 ± 0.04	−0.3 ± 0.3	−0.15 ± 0.01	−0.064 ± 0.007	5 ± 4
Bangladesh^b^	April	0.82 ± 0.05	−0.24 ± 0.09	−0.18 ± 0.01	0.026 ± 0.006	na
Mexico	May	2.75 ± 0.06	−0.68 ± 0.07	0.01 ± 0.01	0.095 ± 0.006	na
Russia	April	4.18 ± 0.07	−1.4 ± 0.2	−0.39 ± 0.02	−0.074 ± 0.003	19 ± 3
Japan^b^	April	4.0 ± 0.3	−1.9 ± 0.2	−0.19 ± 0.02	−0.24 ± 0.04	8 ± 2
Egypt^d^	May	3.1 ± 0.1	−0.4 ± 0.2	−0.03 ± 0.01	−0.25 ± 0.09	1.4 ± 0.9
Iran^d^	April	2.76 ± 0.07	−0.5 ± 0.7	0.080 ± 0.008	−0.12 ± 0.02	4 ± 6
Turkey^d^	April	4.23 ± 0.08	−1.5 ± 0.7	0.17 ± 0.03	0.135 ± 0.007	na
Germany	March	7.95 ± 0.3	−2.7 ± 0.4	−0.77 ± 0.01	−0.12 ± 0.01	23 ± 4
Thailand^b^	March	1.34 ± 0.08	−0.25 ± 0.03	−0.052 ± 0.008	−0.003 ± 0.008	100 ± 200
France	April	4.76 ± 0.03	−3.1 ± 0.1	−0.117 ± 0.008	−0.168 ± 0.009	19 ± 1
United Kingdom	April	6.42 ± 0.03	−2.8 ± 0.1	−0.19 ± 0.02	−0.43 ± 0.01	6.7 ± 0.3
Italy	February	10.9 ± 0.3	−2.8 ± 0.3	−2.84 ± 0.05	−0.37 ± 0.02	8 ± 1
South Africa^d^	May	7.7 ± 0.1	−2.7 ± 0.3	−0.06 ± 0.02	−0.4 ± 0.2	7 ± 3
Spain	April	3.16 ± 0.04	−2.1 ± 0.1	−0.113 ± 0.006	−0.169 ± 0.009	12.6 ± 0.9
Argentina^c^	April	1.63 ± 0.07	−0.8 ± 0.7	−0.32 ± 0.02	−0.08 ± 0.01	11 ± 10
Africa^d^	May	0.66 ± 0.02	−0.15 ± 0.02	−0.012 ± 0.001	−0.051 ± 0.007	2.9 ± 0.6
Asia^b^	March	3.0 ± 0.1	−0.70 ± 0.05	0.002 ± 0.001	−0.19 ± 0.03	3.6 ± 0.6
East Asia^b^	February	6.4 ± 0.1	−1.86 ± 0.02	−0.068 ± 0.001	−0.55 ± 0.06	3.4 ± 0.4
South Asia^b^	June	0.98 ± 0.06	−0.28 ± 0.03	−0.044 ± 0.001	0.015 ± 0.006	na
Europe	April	3.87 ± 0.02	−1.67 ± 0.08	−0.096 ± 0.001	−0.090 ± 0.007	19 ± 2
West Europe	April	4.52 ± 0.02	−2.08 ± 0.07	−0.115 ± 0.001	−0.163 ± 0.009	12.8 ± 0.9
Central Europe	April	2.86 ± 0.05	−1.0 ± 0.2	0.013 ± 0.001	0.053 ± 0.005	na
East Europe	April	3.43 ± 0.03	−1.40 ± 0.06	−0.167 ± 0.001	−0.049 ± 0.004	29 ± 2
North America	April	2.41 ± 0.07	−0.5 ± 0.1	−0.105 ± 0.001	−0.029 ± 0.008	17 ± 7
Oceania	May	1.59 ± 0.09	−0.2 ± 0.1	−0.024 ± 0.001	−0.086 ± 0.005	2 ± 2
South America^c^	April	1.11 ± 0.05	−0.4 ± 0.4	−0.022 ± 0.001	−0.056 ± 0.007	8 ± 7
Global (country level)	April	1.5 ± 0.2	−0.53 ± 0.06	−0.050 ± 0.010	−0.04 ± 0.01	15 ± 4
Global (population-weighted)	April	2.2 ± 0.5	−0.52 ± 0.08	−0.06 ± 0.04	−0.10 ± 0.05	5 ± 3

Countries with largest populations and annual mean population-weighted NO_2_ concentrations greater than 1 ppbv are shown for months with the greatest 2020–2019 difference and strict lockdown conditions (stringency index >20), sorted by population. Regional and global data also shown.

^a^Satellite-inferred annual mean ground-level NO_2_ trends are scaled by the ratio of the 2019 monthly mean to the annual mean to account for seasonality.

Long-term country-level trends are calculated for 2005–2019, except for countries/regions in:

^b^Asia: 2013–2019.

^c^South America: 2011–2019.

^d^Africa and the Middle East: 2015–2019.

na, Ratio of 2020–2019 difference to long-term trend not calculated when one value is positive and one is negative.

Meteorological differences are calculated with the GEOS-Chem chemical transport model using emission inventories that do not include changes that occurred owing to COVID-19 lockdown policies but do reflect meteorological changes. Supplementary Fig. 10 shows TROPOMI-derived changes at 2.0° × 2.5° resolution for comparisons with simulated values at the same resolution. Population-weighted NO_2_ concentration changes due to meteorology in Asia, Europe, South America, Africa and the Middle East are a factor of 2–6 smaller than observed; thus, meteorology alone cannot explain the observed decreases. Concentration increases in the central USA, as noted in other studies^10^, do not appear to be meteorologically driven and may be due to changes in biogenic NO_*x*_ sources.

Supplementary Fig. 11 shows the ratio of population-weighted January–June monthly mean NO_2_ concentrations in 2020 to 2019 across selected regions. Most regions have the largest decrease in NO_2_ in April when lockdown conditions were strongest (the global mean COVID restriction stringency index (defined in Methods) reached a maximum of 0.79 on 18 April), apart from China, where lockdowns were initiated in January. In most regions, 2020 NO_2_ concentrations return towards pre-lockdown values in May or June owing to relaxing travel restrictions (30 June global mean stringency index, 0.60) as well as increasing soil, lightning and biomass-burning emissions that lessen the sensitivity of ambient NO_2_ to anthropogenic emissions.

## City- and country-level NO_2_ changes

The fine resolution of our satellite-derived ground-level NO_2_ dataset enables the assessment of larger changes in NO_2_ concentrations from 2020–2019 evident at the city level. We calculate changes in TROPOMI-observed monthly mean ground-level NO_2_ from 2020–2019 over 215 major cities (the ten most populous cities in each country with a population greater than 1 million) for the month with the greatest monthly mean lockdown stringency index, compared with expected changes due to meteorology and long-term trends (Supplementary Table 1). Most cities have TROPOMI-derived NO_2_ decreases that cannot be explained by changes due to meteorology alone. For example, satellite-derived NO_2_ concentrations in Beijing decreased by 45% in March, despite meteorological conditions favourable to increased NO_2_. Jakarta, Manila, Istanbul, Los Angeles and Buenos Aires among others had decreased NO_2_, despite similarly unfavourable meteorological conditions. Some cities, including Moscow, Tokyo, London, New York, Toronto and Delhi, had meteorological conditions that would have led to NO_2_ decreases regardless of emission changes, but observed concentration changes exceeded the expected meteorological change.

Consistent analysis of individual cities as enabled by this dataset reveals a mean observed decrease of 32 ± 2% for these 215 cities. The mean expected meteorologically driven change was −1 ± 1% and the mean expected change owing to long-term trends was a decrease of 1.4 ± 0.4%. Supplementary Fig. 12 shows these reductions to be consistent with those found in 381 ground-monitor values from 79 studies^34^ (32 ± 2%). Of the 215 cities included here, 65 are in countries that did not have ground-monitoring data available for previous studies. Notably, the 65 cities without monitors are largely in lower-income countries of Africa and southeast Asia. The average gross national income per capita for unmonitored countries is US$7,100, compared to US$25,000 for monitored countries, illustrating the potential of satellite-derived ground-level concentrations for providing information about lower-income regions. In summary, the observed decreases in NO_2_ across more than 200 cities worldwide were generally driven by COVID-19 lockdowns, with locally varying modulation by meteorology and business-as-usual changes.

Table 1 shows monthly mean country-level population-weighted NO_2_ concentrations, changes during COVID-19 lockdown restrictions, meteorological effects and long-term trends for the month with the greatest 2020–2019 change. Meteorological effects were generally minor at the national and regional scale. Multi-year trends provide context for the scale of the changes observed during COVID-19 lockdowns. The decrease in March NO_2_ concentrations in the USA from 2019 to 2020 was equivalent to four years of long-term NO_2_ reductions. Similarly, changes in NO_2_ during COVID-19 lockdowns were equivalent to greater than three years of reductions in China, and up to 23 years in Germany. Globally, the April 2020 population-weighted NO_2_ concentration was 0.53 ± 0.06 ppbv lower than in April 2019, equivalent to 15 ± 4 years of global NO_2_ reductions.

## NO_2_ as a lockdown indicator

The relationship between this satellite-derived ground-level NO_2_ dataset and lockdown stringency provides supporting evidence for the impact of travel restrictions (Supplementary Fig. 13). The ratio of population-weighted mean observed NO_2_ in 2020 to 2019 was calculated for each country and each month from January to June. The 2020/2019 NO_2_ ratio in countries with the strictest lockdown (monthly minimum stringency indices greater than the 75th percentile) was 29 ± 3% lower than for countries with the weakest lockdowns (monthly median stringency indices less than the 25th percentile). Maximum and median ratios were also lower for countries with strict lockdowns. Both distributions have similar variability (standard deviations 0.02 and 0.03) which demonstrates similar interannual variability due to meteorology for both sets. When focusing on only the month with the strictest lockdown for each country, changes in population-weighted NO_2_ are correlated with lockdown intensity, with changes in countries with strict lockdowns (average decrease 43% if lockdown index >80) more than three times as large as in those with weaker lockdowns (12% if lockdown index <40).

This relationship suggests that changes in satellite-derived NO_2_ concentrations offer observational information on the spatial distribution of lockdown effects that is not available through policy-based stringency indices. For example, although the policy-based stringency index in most cases provides a single value for a country, city-level NO_2_ concentration decreases in India are in the range 30–84%, reflecting variability in local mobility restrictions, emissions sources, and their sensitivity to lockdowns. Supplementary Fig. 14 explores the sensitivity of NO_2_ concentrations to emissions from the transportation and electricity sectors in India, China and the USA by examining the distribution of changes in NO_2_ concentration at the 20 largest population centres and 20 largest fossil fuel-burning power plants in each country. All countries have substantial NO_2_ decreases in cities, but the sensitivities vary in areas associated with the electricity sector, with decreasing concentrations near power plants in India (mean change −35 ± 4%) and China (−28 ± 8%) but insignificant changes in the USA (−4 ± 8%). Observed NO_2_ changes at these power plants exceed expected changes from meteorology alone (−8 ± 2%, −1 ± 4% and −1 ± 3% in India, China and the USA, respectively). Although variability between power plants reflects a mix of regionally varying factors, including meteorology, electricity demand, fuel type and plant-specific emission controls, as well as changes in nearby emissions from other sectors including transportation, these differences indicate a sensitivity of local air quality to activity restrictions affecting the energy sector.

Examining geographic differences in satellite-derived NO_2_ concentrations within metropolitan regions is also informative. For example, variability between emission sources is apparent around the city of Atlanta, Georgia, USA (Supplementary Fig. 15). The population-weighted NO_2_ concentration in Atlanta and the surrounding region dropped by 28% from April 2019 to 2020, but with substantial spatial variability in the observed change. The greatest NO_2_ decreases are found near a large coal-powered electricity plant to the southeast of the city, with significant changes near another plant to the northwest. Decreases were also larger near the Hartsfield–Jackson International Airport—reflecting the dramatic slowdown in air travel—and over suburban regions to the west and northeast of the city centre, than in the downtown core. Supplementary Fig. 15 also demonstrates the range of NO_2_ changes experienced by the local population. Over 1.2 million people live in regions where NO_2_ decreases exceeded 40%, whereas nearly 1 million people experienced decreases of 10% or less. Similar heterogeneity in population exposure exists in other major cities, as demonstrated by Supplementary Fig. 16. For example, a subset of over 1 million people in the Paris metropolitan area experienced NO_2_ decreases of 75% (4.5 ppbv) or more (10th-percentile exposure), whereas another similar-sized subset experienced changes of 23% (0.6 ppbv) or less (90th-percentile exposure). Of the cities examined here, 68 had an interquartile range in population exposure change during lockdowns of 20 percentage points or larger, 22 of which were unmonitored cities. Studies have found that NO_2_ changes during lockdowns varied among socioeconomic, ethnic and racial groups in US cities^46^, and thus the variability in other major cities observed here suggest similar disparities may occur elsewhere. The heterogeneity of NO_2_ changes demonstrates the need for the finely resolved information on lockdown effects that is offered by satellite observations.

We find that using this satellite-derived NO_2_ dataset as an observational proxy for lockdown conditions is also useful for identifying links between lockdown-driven emission changes and secondary pollutants. For example, several studies have found little to no change in fine particulate matter (PM_2.5_) during lockdowns as meteorology, long-range transport and nonlinear chemistry complicate the relationship between PM_2.5_ and NO_*x*_ emissions^47,48^. A challenge in these studies has been limited observational information on the local lockdown intensity. Recent work examining 2020–2019 changes in satellite-derived PM_2.5_ concentrations found that lockdown-driven decreases in PM_2.5_ concentration can be identified by separating the meteorological effects from emissions effects using chemical transport modelling and focusing on regions with the greatest sensitivity to emission reductions^49^. Here we examine that same satellite-derived PM_2.5_ dataset using TROPOMI-derived ground-level NO_2_ concentrations to identify the regions where PM_2.5_ concentrations are most likely associated with lockdowns or sensitive to NO_*x*_ emissions. Supplementary Fig. 17 shows the distribution of changes in monthly mean PM_2.5_ concentrations from 2020–2019 for China in February and North America and Europe in April. Regions with the largest 2020–2019 NO_2_ concentration decreases (90th percentile) are considered to be those with significant NO_*x*_ emission reductions. Population-weighted mean PM_2.5_ concentrations decreased overall; however, regions with the largest NO_2_ decreases experienced greater local changes in PM_2.5_ concentration in China and to a lesser extent in North America, indicating that the sensitivity of PM_2.5_ to changing NO_*x*_ emissions can be inferred. The year-to-year variability of PM_2.5_ concentrations in Europe is similar regardless of changes in NO_2_, indicating a greater role of meteorology or transport on PM_2.5_ in this region and period. These results are consistent with previous findings when using chemical transport modelling to identify regions where local emissions are important^49^. Thus, the observational proxy on lockdown conditions offered by these satellite-derived surface NO_2_ concentrations offers spatially resolved information to identify where PM_2.5_ and NO_2_ (and by proxy, NO_*x*_ emissions) are most strongly coupled.

## Implications

The pronounced decreases in ground-level NO_2_ found here for over 200 cities worldwide during COVID-19 lockdowns are a culmination of recent advancements in techniques for estimating ground-level NO_2_ from satellite observations^27^ alongside higher-resolution satellite observations from TROPOMI that allow for estimating high spatial resolution, short-term changes in NO_2_ exposure. This method bridges the gap between monitor data (that measure ground-level air quality but have poor spatial representativeness) and satellite column data (that provide spatial distributions but are less representative of ground-level air quality). The ability to infer global ground-level NO_2_ concentrations with sufficient resolution to assess individual cities and even within-city gradients is an important development in satellite remote-sensing instrumentation and algorithms. Additionally, these satellite-derived ground-level NO_2_ concentrations offer information about unmonitored communities and populations that are underrepresented in studies focused on ground-monitor data. These cities are found to have different characteristics of NO_2_ concentrations and changes during lockdowns that motivate the need for satellite observations in the absence of local ground monitoring. The changes in ground-level NO_2_ due to COVID-19 lockdown restrictions, which exceed recent long-term trends and expected meteorologically driven changes, demonstrate the impact that policies that limit emissions can have on NO_2_ exposure. This information has relevance to health impact assessment; for example, studies focused on ground-monitor data have indicated improvements in health outcomes related to improved air quality during lockdowns, including an estimated 780,000 fewer deaths and 1.6 million fewer paediatric asthma cases worldwide due to decreased NO_2_ exposure^20^. Our study demonstrates considerable spatial variability in lockdown-driven ground level NO_2_ changes that does not necessarily correlate with population density, demonstrating probable uncertainties arising from extrapolating changes observed by ground monitors to estimate broad changes in population NO_2_ exposure. Satellite-based ground-level NO_2_ estimates provide high-resolution information on the spatial distribution of NO_2_ changes in 2020 that cannot be achieved through ground monitoring, particularly in regions without adequate ground monitoring, and should improve exposure estimates in future health studies. Additionally, ground-level concentrations from downscaled OMI observations provide the opportunity to contrast effects of past mitigation efforts on long-term NO_2_ trends against the short-term changes resulting from more dramatic regulations, and a chance to improve studies of health outcomes related to long-term NO_2_ exposure.

The strength of the links between observed changes in NO_2_ concentration and lockdown stringency indicates that satellite-based ground-level NO_2_ concentrations offer useful observational, spatially resolved information about lockdown conditions. This provides an observational metric for examining the efficacy of lockdown restrictions on restricting mobility for studies examining the spread of COVID-19. Here we exploited this information to illustrate the differing sensitivity of NO_2_ concentrations to changes in various emission sources to lockdown restrictions. Future applications of these data could include examining socioeconomic drivers that impact this variability within and between countries. Comparisons between satellite-derived ground-level NO_2_ and PM_2.5_ also indicate the utility of these data as an observational proxy for identifying regions where secondary pollutants such as PM_2.5_ or ozone are more likely to be sensitive to NO_*x*_ emissions; these links are otherwise difficult to trace without the use of chemical transport models^50^.

These data offer information to improve NO_2_-exposure estimates, to examine exposure trends, and subsequently estimate changes in health burden. These developments provide an excellent opportunity for advances in air quality health assessment in relation to NO_2_ and its combustion-related air pollutant mixture.

## Methods

### Data

We use tropospheric NO_2_ columns from the OMI (NASA Standard Product version 4)^51^ and TROPOMI^52,53^ satellite instruments. Both instruments measure solar backscatter radiation in the ultraviolet–visible (UV–vis) spectral bands on sun-synchronous orbits with local overpass times around 1:30 p.m. TROPOMI observations from April 2018–October 2020 are used to examine near-term NO_2_, and OMI observations from January 2005–December 2019 are used to examine long-term trends. Observations with retrieved cloud fractions greater than 0.1 or flagged as poor quality or snow-covered (that is, TROPOMI quality assurance flag <0.75) are excluded. Although the resolution of TROPOMI observations is 3.5 × 5.5 km^2^, several studies have demonstrated that oversampling techniques can provide accurate NO_2_ maps at 1 × 1 km^2^ resolution when averaging over a one-month period^31,32,54^. An area-weighted oversampling technique^55,56^ is used to map daily satellite NO_2_ column observations from TROPOMI onto a ~0.01° × 0.01° (~1 × 1 km^2^) resolution grid and from OMI to a 0.1° × 0.125° (~10 × 10 km^2^) grid, as these resolutions balance the need of fine resolution for observing fine-scale structure and of minimizing the effects of sampling biases and noise in the observations. Supplementary Fig. 8 provides further evidence that a one-month period provides sufficient observations for a 1 × 1 km^2^ map as the agreement between TROPOMI-derived surface concentrations and in situ observations does not deteriorate when the sampling period is reduced from one year to one month. Additionally, we compared 2019 monthly mean concentration estimates with the 2019 annual mean and find high correlation (*r* = 0.90), indicating similar spatial variability. We correct for sampling biases in the satellite records due to persistent cloudy periods or surface snow cover using a correction factor calculated with the GEOS-Chem chemical transport model described below by sampling the GEOS-Chem-simulated monthly or annual mean column densities to match the satellite.

We use hourly ground-level NO_2_ measurements from monitors to constrain and evaluate the satellite-based estimates. Observations from the US Environmental Protection Agency Air Quality System (https://aqs.epa.gov/aqsweb/documents/data_mart_welcome.html) over the continental USA from 2005–2020, Environment and Climate Change Canada’s National Air Pollution Surveillance Program (http://maps-cartes.ec.gc.ca/rnspa-naps/data.aspx) from 2005–2019, European Environment Agency (https://aqportal.discomap.eea.europa.eu/index.php/users-corner/) from 2005–2020, National Air Quality Monitoring Network in China from 2015–2020 were (obtained from https://quotsoft.net/air) were used. European monitors classified as near-road are excluded. Monthly and annual mean concentrations at each site are calculated by averaging hourly observations between 13:00–15:00 h (corresponding to satellite overpass times) and corrected for the known overestimate in regulatory measurements due to interference of other reactive nitrogen species following Lamsal et al.^24^.

To examine the relationship between COVID-19 lockdown policies and ground-level NO_2_ concentrations, we use the Oxford COVID-19 Government Response Tracker (OxCGRT, https://www.bsg.ox.ac.uk/research/research-projects/coronavirus-government-response-tracker#data). OxCGRT provides a daily country-level policy ‘stringency index’ ranging from 0–100 that is based on containment and closure policies (for example, school and workplace closures, stay-at-home orders, gathering restrictions). We also use population density data from the Center for International Earth Science Information Network for the available years of 2005, 2010, 2015 and 2020, and linearly interpolate for other years (10.7927/H4JW8BX5).

### Inferring ground-level concentrations from satellite column observations

Ground-level NO_2_ concentrations are derived from TROPOMI NO_2_ columns following the method developed in Cooper et al.^27^. This algorithm builds upon the method first developed by Lamsal et al.^24^ which uses the GEOS-Chem-simulated relationship between ground-level and tropospheric column NO_2_ concentrations. The updated algorithm uses the satellite-observed column densities and ground-monitor data as observational constraints on the shape of the boundary layer profile, reducing the sensitivity to model resolution and improving agreement between satellite-derived ground-level concentrations and in situ observations. Technical details on the application of this method as used here are available in the Supplementary Information.

For long-term trend analysis, we use more recent TROPOMI observations to provide fine-resolution spatial structure to the OMI-observed NO_2_ columns following the method of Geddes et al.^25^. Annual mean OMI NO_2_ columns are gridded to 10 × 10 km^2^ resolution and a median-value filter is applied to reduce noise. We smooth the two-year (April 2018–April 2020) mean TROPOMI NO_2_ columns mapped at 1 × 1 km^2^ resolution using a two-dimensional boxcar algorithm with an averaging window of 10 × 10 km^2^ to match the resolution of the gridded OMI NO_2_ columns. We then downscale the annual mean OMI NO_2_ columns using the ratio of the 1 × 1 km^2^ TROPOMI columns to the smoothed TROPOMI columns. The downscaled columns are then used to infer ground-level concentrations following the method used for TROPOMI. Supplementary Fig. 18 demonstrates the utility of this downscaling approach by comparing OMI-derived ground-level concentrations to those derived from the downscaled columns. When comparing 2020–2019 changes in monthly mean concentrations to long-term trends, trends in annual mean concentration are scaled by the ratio of the 2019 monthly mean to the 2019 annual mean to account for seasonality.

The GEOS-Chem chemical transport model version 11-01 is used here (https://geos-chem.seas.harvard.edu/) for NO_2_ vertical profiles and to assess meteorological effects. GEOS-Chem simulates atmospheric chemistry and physics using a detailed HO_*x*_–NO_*x*_–VOC–O_3_–aerosol chemical mechanism^57,58^ driven by meteorological data from the MERRA-2 Reanalysis of the NASA Global Modeling and Assimilation Office^59^. A detailed description of the simulation is provided in Hammer et al.^60^. We replace the a priori profile used in the retrieval with profiles simulated using the GEOS-Chem model to ensure consistency in vertical profile representation between TROPOMI, OMI, and GEOS-Chem. We simulate NO_2_ profiles from January 2005–June 2020 at a horizontal resolution of 2° × 2.5°. Supplementary Fig. 19 shows results from tests using a simulation at 0.5° × 0.625° which was available over North America, Europe and Asia. Satellite-derived ground-level concentrations at ~1 × 1 km^2^ resolution were not sensitive to the resolution of the a priori information, consistent with Cooper et al.^27^, and thus the 2° × 2.5° was used here for consistency across all regions.

### Inferring country- and city-level NO_2_ changes during COVID lockdowns

City-level monthly means are calculated from TROPOMI-derived concentrations at ~1 × 1 km^2^ resolution averaged over a 20 × 20 km^2^ region surrounding the city. Meteorological effects are estimated using GEOS-Chem simulations at 2° × 2.5° resolution with consistent emissions in both years, downscaled to ~1 × 1 km^2^ resolution using the horizontal variability of TROPOMI-derived ground-level concentrations. Supplementary Fig. 20 demonstrates that GEOS-Chem simulations can represent meteorologically driven changes in NO_2_ in pre-lockdown periods. Trends are defined over 2005–2019 for North America, Europe and Australia, 2015–2019 for Asia and Africa, and 2010–2019 for South America and scaled for seasonality.

Country-level population-weighted means, used to represent population NO_2_ exposure, are calculated using concentrations at ~1 × 1-km^2^ resolution via:

2 $${\rm{population}}-{\rm{weighed}}\,{\rm{mean}}=\frac{{\sum }_{i=1}^{{\rm{grid}}\,{\rm{boxes}}\,{\rm{in}}\,{\rm{country}}}{P}_{i}\,{x}_{i}}{{\sum }_{i=1}^{{\rm{grid}}\,{\rm{boxes}}\,{\rm{in}}\,{\rm{country}}}{P}_{i}},$$

where *x*_*i*_ is the NO_2_ concentration and *P*_*i*_ is the population within a ~1 × 1-km^2^ grid box.

### Limitations and sources of uncertainty

Uncertainty values for country- and region-level population-weighed means (*σ*_total_) represent the sum in quadrature of three main error sources:

3 $${\sigma }_{{\rm{total}}}=\sqrt{{\sigma }_{{\rm{pop}}-{\rm{weighted}}}^{2}+{\sigma }_{{{\Omega }}_{{\rm{\max }}}}^{2}+{\sigma }_{{\rm{AMF2020}}}^{2}}.$$

Uncertainty in population-weighted means (*σ*_pop-weighted_) are estimated using a bootstrapping method^61^. Uncertainty in 2020 NO_2_ estimates (*σ*_AMF2020_) arises from the use of simulated profiles as a priori information for calculating satellite air mass factors and for informing the column-to-ground-level relationship, as these simulations use emission inventories that do not reflect changes resulting from COVID-19-related travel restrictions. Such errors may result in overestimating the fraction of columnar NO_2_ near the surface, resulting in an overestimate in satellite-derived ground-level NO_2_ concentrations and an underestimate of the 2020–2019 difference. We estimate *σ*_AMF2020_ by performing sensitivity studies where anthropogenic NO_*x*_ emissions were uniformly reduced by 50% to assess the effect of such emission errors on ground-level NO_2_ estimates. Reducing anthropogenic NO_*x*_ emissions by 50% led to a 5% change in monthly mean population weighted NO_2_ concentrations in North America, Europe and Asia for March 2020. Aerosols can also contribute to uncertainty in air mass factor calculations, as a reduction in anthropogenic scattering aerosols during lockdowns may reduce air mass factors leading an underestimation of the NO_2_ change^62,63^. However, this is likely to be a minor source of uncertainty in estimated NO_2_ changes due to lockdown, because aerosol concentration changes were small in most regions^49^ and a reduction in aerosol concentration of 10% translates to an uncertainty in NO_2_ of less than 5%^64^. Additional uncertainty (${\sigma }_{{{\Omega }}_{\max }}$) may arise from the choice of the *Ω*_max_ parameter (described in the Supplementary Information), particularly in regions where there are insufficient ground-monitor data for constraining *Ω*_max_. We estimate ${\sigma }_{{{\Omega }}_{\max }}$ by evaluating the sensitivity of mean population-weighted NO_2_ concentrations to a 20% change in *Ω*_max_. Median country-level ${\sigma }_{{{\Omega }}_{\max }}$ values are ~7%. Uncertainty values in trends are calculated by a weighted linear regression where annual mean concentrations are weighted by *σ*_total_.

Although tests here indicate that satellite-derived ground-level NO_2_ concentrations are insensitive to the resolution of the simulated data used in the algorithm, discontinuities can occur at the edges of simulation grid boxes. To quantify this uncertainty, we calculate the difference across the grid box boundaries in each region. In most regions the discontinuity is small (<0.5 ppbv in 92% of total cases, and in 98% of cases where NO_2_ concentrations >2 ppbv) although can be larger in some cases (>2 ppbv in 0.02% of cases where NO_2_ concentrations >2 ppbv, maximum of 4.5 ppbv).

The along-track resolution of TROPOMI observations changed from 7 km to 5.5 km in August 2019. This change may influence interannual comparisons, particularly with respect to the sub-grid downscaling of process which relies on the spatial structure observed by the satellite. To test the influence of this change, we perform a case study where annual mean surface concentrations over Asia are calculated using two different sub-grid scaling factors (*ν* in equation S1 in the Supplementary Information) determined from one year of observations before and after the resolution change, with other variables held constant. The mean relative difference between the two tests was 9% for grid boxes with annual mean concentrations greater than 1 ppbv, with a change in regional population-weighted NO_2_ concentrations of 3%. Greater sensitivity to observation resolution was evident in regions with larger NO_2_ enhancements, although relative differences greater than 25% occur in fewer than 5% of grid boxes. These tests indicate that although the change in observation resolution may change some spatial gradients, the overall impact on population exposure estimates is small.

Uncertainty values presented above represent uncertainty in the conversion of satellite-observed slant columns into surface concentrations and do not represent systematic errors in the retrieval of slant columns from satellite-observed radiances (~10%), or errors in the air mass factor calculations (23–37%), both of which have been extensively examined in prior studies^52,65^. Errors related to air mass factor calculations can be reduced by using higher-resolution inputs in air mass factor calculations^66,67^ and are partially mitigated here during the conversion of column densities to surface concentrations through the sub-grid parameterization^27^.

Although we apply a scaling factor to correct for sampling biases due to persistent cloud cover or surface snow cover, biases in monthly mean calculations may persist if the sampling rate is sufficiently low, particularly for city-level calculations. Most of the cities examined in Supplementary Table 1 had sufficient sampling to allow for a robust monthly mean calculation (median sampling rate of 14 days per month for the months indicated in the table), except for two cities for which fewer than 5 days of observations per month were available for the given month in either 2019 or 2020 (labelled * in Supplementary Table 1). However, results from these cities were consistent with nearby, more frequently sampled cities, lending confidence to these results despite the lower sampling frequency.

This dataset represents substantial improvement over past satellite-derived ground-level NO_2_ estimates, as the updated algorithm is less sensitive to model resolution and leverages higher-resolution satellite observations than previous estimates. However, limitations remain. There can be considerable fine-scale variability at scales finer than the 1 × 1 km^2^ resolution used here that cannot be captured by the satellite observations^68,69^. Additionally, ground-monitor data are used as a constraint in converting observed column densities to ground-level concentrations, and thus absolute concentration values are probably less accurate in time periods or regions where ground-monitor data are unavailable. However, these data are still useful for examining relative interannual variability or trend analysis. In combining OMI and TROPOMI observations we assume that the spatial gradients observed by TROPOMI in 2018–2020 can be applied to OMI for the entire 2005–2019 time series. New or disappearing point emission sources with small plume footprints may affect this assumption; however, past evaluations of similar assumptions have not found it to be a substantial error source^25^. Additional errors in the column to ground-level conversion may occur in areas with substantial free tropospheric NO_2_ sources such as aircraft emissions or lightning.

## Online content

Any methods, additional references, Nature Research reporting summaries, source data, extended data, supplementary information, acknowledgements, peer review information; details of author contributions and competing interests; and statements of data and code availability are available at 10.1038/s41586-021-04229-0.

## Supplementary information

Supplementary Information This file contains Supplementary Methods, Supplementary Table 1, Supplementary Figures 1–20, and additional references.

## Data Availability

TROPOMI-derived 2019 annual mean ground-level NO_2_ concentrations developed here are available at 10.5281/zenodo.5484305. TROPOMI-derived January–June 2019 and 2020 concentrations are available at 10.5281/zenodo.5484307. Satellite-derived ground-level NO_2_ concentrations for 2005–2019 used for trend analysis are available at 10.5281/zenodo.5424752. Satellite column data used here are available from the NASA Goddard Earth Sciences Data and Information Services Center (TROPOMI, 10.5270/S5P-s4ljg54; OMI, 10.5067/Aura/OMI/DATA2017). The GEOS-Chem model version used here is available at 10.5281/zenodo.2658178. Hourly ground-level NO_2_ measurements from ground monitors in the USA are available from the US Environmental Protection Agency Air Quality System (https://aqs.epa.gov/aqsweb/documents/data_mart_welcome.html), in Canada from Environment and Climate Change Canada’s National Air Pollution Surveillance Program (http://maps-cartes.ec.gc.ca/rnspa-naps/data.aspx), in Europe from the European Environment Agency (https://aqportal.discomap.eea.europa.eu/index.php/users-corner/), and in China from https://quotsoft.net/air. COVID-19 lockdown policy information is provided by the Oxford COVID-19 Government Response Tracker (https://www.bsg.ox.ac.uk/research/research-projects/coronavirus-government-response-tracker#data). Population distribution data are available from the Center for International Earth Science Information Network, 10.7927/H4JW8BX5. NO_2_ changes during COVID-19 lockdowns from previous studies used for comparison here were compiled by Gkatzelis et al.^34^ and are available at https://covid-aqs.fz-juelich.de. Gross National Income data were provided by World Bank, available at https://data.worldbank.org/indicator/ny.gnp.pcap.cd?year_high_desc=true.

## Code availability

Code used to calculate surface NO_2_ concentrations from satellite columns is available upon request. Some features in the displayed maps were produced using The Climate Data Toolbox for MATLAB^70^.
