# Supplementary material for: Global fine-scale changes in ambient NO2 during COVID-19 lockdowns
Source: Nature. 2022 Jan 19;601(7893):380–7. doi: 10.1038/s41586-021-04229-0 (PMC8770130; doi:10.1038/s41586-021-04229-0)
Supplement: Supplementary file 1 — This file contains Supplementary Methods, Supplementary Table 1, Supplementary Figures 1–20, and additional references. [file 41586_2021_4229_MOESM1_ESM.pdf]

nature portfolio

https://doi.org/10.1038/s41586-021-04229-0

Supplementary information

Global fine-scale changes in ambient NO_2 during COVID-19 lockdowns

In the format provided by the authors and unedited

Supplemental Material

Methods: Inferring surface concentrations

We calculate satellite-derived surface NO_2 concentrations S^o_s from satellite column observations by further developing the method described in Cooper et al.[1]:

$$S_S^o = \kappa \left(\frac{\nu\Omega - \Omega^f}{\Omega^b}\right)\left(\frac{S}{\Omega}\right)\overline{\Omega^o} \quad \text{(S1)}$$

where the surface concentration S and tropospheric Ω, free tropospheric Ω^f, and boundary layer Ω^b vertical column densities are simulated by the GEOS-Chem chemical transport model, $\overline{\Omega^o}$ is the average satellite tropospheric column within the model grid box, and ν represents the satellite-observed sub-model-grid spatial variability. The parameter κ allows the satellite column densities to constrain the shape of the boundary layer profile, and is defined as:

$$\kappa = \begin{cases} (1-w) + \chi w & if\ \Omega_o < \Omega_{max} \\ \chi & if\ \Omega_o > \Omega_{max} \end{cases} \quad \text{(S2)}$$

where

$$w = \frac{\Omega_o}{\Omega_{max}} \quad \text{(S3)}$$

Ω_{max} is used to distinguish between clean and polluted regions, and χ is a scaling factor that corrects for biases in the algorithm over regions with large surface emission sources. In Cooper et al[1] χ is calculated for each model grid box

$$\chi = \frac{\max\,(S_S^o\ assuming\ all\ NO_2\ in\ the\ surface\ layer)}{\max\,(S_S^o\ assuming\ NO_2\ well\ mixed\ in\ boundary\ layer)} \quad \text{(S4)}$$

In this paper we calculate χ using the 95th percentile values within a grid box instead of the maximum values to reduce influence of noise in the satellite observations.

The scaling factor χ includes a factor ν defined as

$$\nu = \frac{\Omega_o}{\overline{\Omega^o}} \quad \text{(S5)}$$

In this paper we place an upper limit of 3 on ν to avoid amplifying spurious signals.

Ω_{max} is selected to minimize error between satellite-derived surface concentrations and ground monitor data. In Cooper et al[1] Ω_{max} was 11x10^{15} molec/cm^2. In this paper, Ω_{max} is adjusted for each year (for annual means) or month (for monthly means) in each region based on available ground monitor data. We use the Ω_{max} values for Asia in 2015 for 2005-2014 estimates in that region as ground monitor data were unavailable. Sufficient ground data for constraining Ω_{max} are not available in South America, Africa, the Middle East, or Australia. In these regions, Ω_{max} is set to the average of values used in North America, Europe, and Asia. Tests performed here found that long-term trends change by <1.5 %/year and 2020-2019 differences in population-weighted monthly mean concentrations change by <5 percentage points in these regions for Ω_{max} values ranging from 5-20x10^{15} molec/cm^2, indicating that both trends and interannual changes are not sensitive to the choice of Ω_{max} when the same values are used in each year of the time series.

Supplemental Figure 17 demonstrates that comparison between TROPOMI-derived surface concentrations and those derived from downscaled OMI columns in 2019 indicate differences resulting from biases between TROPOMI- and OMI-observed columns. To correct for this bias, we apply to each year of the OMI timeseries a scaling factor defined by the ratio of TROPOMI-derived surface concentrations to downscaled-OMI-derived surface concentrations in 2019. The scaling factor is applied in grid boxes with TROPOMI-derived surface concentrations greater than 5 ppbv to avoid amplifying spurious values.

Supplemental Tables

Supplemental Table 1: Difference in TROPOMI-derived monthly mean surface NO_2 from 2020-2019 for the ten most populous cities with urban area populations greater than 1 million in each country. Differences derived from TROPOMI observations, expected changes in NO_2 due to meteorology, and long-term trends are shown. The month with greatest monthly mean lockdown stringency index is chosen for each city. Values represent mean concentrations in a 20x20 km^2 region surrounding the city. Meteorological effects are estimated using GEOS-Chem simulations at 2°x2.5° resolution with consistent emissions in both years, downscaled to ~1x1 km^2 resolution using the horizontal variability of TROPOMI-derived surface concentrations. Trends are defined over 2005-2019 for North America, Europe, and Oceania, 2015-2019 for Asia and Africa/Middle East, and 2010-2019 for South America and scaled for seasonality. All cities were observed by TROPOMI on at least 5 days in the given month in both 2019 and 2020, except for two cities (marked with *) where fewer than 5 days of TROPOMI observations were available in either year due to persistent cloud cover. Cities that are not included in previous studies[2] are marked with †.

Region	Month with strictest lockdown	City	Country	SI	Observed Change (%)	Expected change from meteorology (%)	Long-term trend (%)
Asia	Feb	Taipei	Taiwan	28	-21±16	6±2	-4.26±0.03

Region	Month with strictest lockdown	City	Country	SI	Observed Change (%)	Expected change from meteorology (%)	Long-term trend (%)
Asia	Feb	Zhongli	Taiwan	28	-13.4±0.4	-8±2	2.6±0.2
Asia	Feb	Kaohsiung	Taiwan	28	-4±11	-3±2	-3.1±0.1
Asia	Feb	Tainan	Taiwan	28	2±5	-2±3	-3.25±0.09
Asia	Feb	Taichung	Taiwan	28	9±4	-0±2	-4.1±0.1
Asia	Mar	Guangzhou*	China	60	-58±3	8.3±0.5	-8.7±0.1
Asia	Mar	Nanyang	China	60	-53±1	5±1	-1.219±0.009
Asia	Mar	Shanghai	China	60	-45±2	-24.0±0.5	-10.26±0.10
Asia	Mar	Beijing	China	60	-45±1	5.7±0.3	-10.4±0.2
Asia	Mar	Shenzhen	China	60	-40±4	-6±2	-4.7±0.1
Asia	Mar	Shijiazhuang	China	60	-19±2	13.2±0.5	-5.2±0.1
Asia	Mar	Tianjin	China	60	-13±2	5.7±0.5	-3.6±0.1
Asia	Mar	Linyi	China	60	-5.5±0.1	11±2	-6.40±0.06
Asia	Mar	Chengdu	China	60	6±39	2±1	3.97±0.07
Asia	Mar	Baoding	China	60	10.1±0.7	13.2±0.7	0.0±0.1
Asia	Apr	Dhaka	Bangladesh	91	-55±1	-1±2	8.5±0.2
Asia	Apr	Chattogram	Bangladesh	91	-27.3±0.7	-2±2	6.78±0.07
Asia	Apr	Hong Kong†	Hong Kong	66	63±7	-23±2	-4.8±0.1
Asia	Apr	Delhi	India	99	-84.6±0.8	-17±1	1.9±0.1
Asia	Apr	Ahmadabad	India	99	-76±1	-6±2	5.4±0.1
Asia	Apr	Bangalore	India	99	-71.0±0.6	-0±2	-7.0±0.1
Asia	Apr	Surat	India	99	-63±1	-2±1	2.7±0.1
Asia	Apr	Hyderabad	India	99	-62.8±0.8	-15±1	-1.6±0.1
Asia	Apr	Pune	India	99	-61±1	-11±1	5.0±0.1
Asia	Apr	Lucknow	India	99	-57±1	-15.5±0.8	-6.33±0.05
Asia	Apr	Mumbai	India	99	-53±3	0±4	-4.54±0.07
Asia	Apr	Chennai	India	99	-51±4	-1±4	3.06±0.06
Asia	Apr	Kolkata	India	99	-30±4	-2±2	-1.55±0.02
Asia	Apr	Yokohama	Japan	46	-69.1±0.6	-9±1	-13.89±0.05
Asia	Apr	Kawanakajima	Japan	46	-62.1±0.5	-9.2±0.8	-9.58±0.03
Asia	Apr	Kyoto	Japan	46	-54±3	8±2	-7.21±0.08
Asia	Apr	Tokyo	Japan	46	-54±3	-9.2±0.8	-10.64±0.06
Asia	Apr	Kobe	Japan	46	-53±2	8±2	-0.8±0.1
Asia	Apr	Osaka	Japan	46	-45±2	8±1	-6.2±0.2
Asia	Apr	Fukuoka	Japan	46	-44±3	-3±2	-6.3±0.1
Asia	Apr	Saitama	Japan	46	-32±2	-9±2	-6.91±0.03
Asia	Apr	Hiroshima	Japan	46	-21±5	9±2	-12.3±0.1
Asia	Apr	Nagoya	Japan	46	-15±6	31±1	0.97±0.09
Asia	Apr	Nur-Sultan	Kazakhstan	89	-58.8±0.9	-20±1	1.71±0.01
Asia	Apr	Almaty	Kazakhstan	89	-50±16	6±1	3.39±0.05
Asia	Apr	Shymkent	Kazakhstan	89	-25±1	-7±2	-4.53±0.09
Asia	Apr	Lahore†	Pakistan	94	-72±2	-14±1	3.5±0.1

Region	Month with strictest lockdown	City	Country	SI	Observed Change (%)	Expected change from meteorology (%)	Long-term trend (%)
Asia	Apr	Gujranwala[†]	Pakistan	94	-54±4	-14±2	2.2±0.1
Asia	Apr	Faisalabad[†]	Pakistan	94	-53±3	-23±2	-1.2±0.2
Asia	Apr	Rawalpindi[†]	Pakistan	94	-48±4	6±2	2.2±0.2
Asia	Apr	Islamabad[†]	Pakistan	94	-47±4	6±2	1.2±0.2
Asia	Apr	Peshawar[†]	Pakistan	94	-37±5	6±3	0.7±0.2
Asia	Apr	Karachi[†]	Pakistan	94	-16±2	-4±4	-0.0±0.1
Asia	Apr	Manila[†]	Philippines	100	-54±2	11±2	-2.09±0.02
Asia	Apr	Quezon City[†]	Philippines	100	-53±2	11±2	-2.11±0.03
Asia	Apr	Caloocan City[†]	Philippines	100	-52±2	11±1	-1.85±0.03
Asia	Apr	Omsk	Russia	85	-36±4	3±2	-9.18±0.02
Asia	Apr	Krasnoyarsk	Russia	85	9±5	25±2	1.7±0.1
Asia	Apr	Novosibirsk	Russia	85	42±8	85±2	-4.10±0.03
Asia	Apr	Bangkok	Thailand	76	-33±3	12±3	-1.18±0.10
Asia	Apr	Tashkent[†]	Uzbekistan	91	-49±15	-7.3±0.9	-2.47±0.04
Asia	Apr	Hanoi[*†]	Vietnam	88	-58±3	-15±1	3.31±0.07
Asia	Apr	Bien Hoa[†]	Vietnam	88	-15±2	-1±2	0.59±0.05
Asia	Apr	Ho Chi Minh City[†]	Vietnam	88	1±12	1.0±0.7	2.93±0.04
Asia	May	Kabul[†]	Afghanistan	81	-30±5	-12±1	-6.2±0.1
Asia	May	Tangerang[†]	Indonesia	68	-48±2	28±3	1.6±0.1
Asia	May	Semarang[†]	Indonesia	68	-45±4	21±3	3.48±0.05
Asia	May	Bandung[†]	Indonesia	68	-44±5	29±5	-7.28±0.04
Asia	May	Jakarta[†]	Indonesia	68	-34±3	28±4	3.11±0.08
Asia	May	Depok[†]	Indonesia	68	-33±5	28±4	4.48±0.08
Asia	May	Medan[†]	Indonesia	68	-32±5	-3±2	-4.07±0.09
Asia	May	Bekasi[†]	Indonesia	68	-30±2	28±4	1.82±0.07
Asia	May	Bogor[†]	Indonesia	68	-16±7	28±4	1.49±0.09
Asia	May	Surabaya[†]	Indonesia	68	-12±4	31±5	1.3±0.2
Asia	May	Ulaanbaatar[†]	Mongolia	66	-4±1	0±2	3.44±0.01
Asia	May	Singapore	Singapore	75	-8±31	5±2	-1.2±0.1
Europe	Apr	Algiers[†]	Algeria	86	-41±2	-9±2	4.34±0.03
Europe	Apr	Vienna	Austria	79	-30±3	-13.3±0.7	0.28±0.03
Europe	Apr	Baku[†]	Azerbaijan	90	-69±2	16±1	5.51±0.03
Europe	Apr	Minsk[†]	Belarus	16	-48±3	5.7±0.9	0.14±0.02
Europe	Apr	Sofia	Bulgaria	72	-47±3	6±1	0.08±0.03
Europe	Apr	Copenhagen	Denmark	70	-44±4	-1.4±0.9	-0.39±0.02
Europe	Apr	Paris	France	88	-67±1	-9.8±0.4	-3.50±0.03
Europe	Apr	Nice	France	88	-64±2	0±1	-2.32±0.03
Europe	Apr	Tbilisi[†]	Georgia	100	-40±3	-3±1	7.00±0.04
Europe	Apr	Berlin	Germany	77	-33±4	-7.4±0.6	0.32±0.09
Europe	Apr	Munich	Germany	77	-30±2	-5.1±0.8	-3.63±0.08

Region	Month with strictest lockdown	City	Country	SI	Observed Change (%)	Expected change from meteorology (%)	Long-term trend (%)
Europe	Apr	Cologne	Germany	77	-16±1	-11.9±0.8	-2.34±0.02
Europe	Apr	Hamburg	Germany	77	6±4	-14.6±0.8	0.6±0.1
Europe	Apr	Budapest	Hungary	77	-20±3	-9±1	-0.07±0.03
Europe	Apr	Tabriz	Iran	55	-22±25	3±3	6.01±0.07
Europe	Apr	Qom	Iran	55	-17±4	3±1	3.53±0.04
Europe	Apr	Karaj	Iran	55	-10±11	6.9±1.0	4.73±0.06
Europe	Apr	Mashhad	Iran	55	19±56	12.2±0.4	4.12±0.09
Europe	Apr	Mosul	Iraq	93	-42±2	6±3	5.80±0.03
Europe	Apr	Baghdad	Iraq	93	-15±31	11.6±0.1	10.3±0.1
Europe	Apr	Dublin	Ireland	90	-28±5	-30.4±1.0	-2.90±0.02
Europe	Apr	Rome	Italy	93	-65±2	6±2	-1.18±0.04
Europe	Apr	Milan	Italy	93	-44.6±0.8	-12.6±0.7	-3.00±0.05
Europe	Apr	Irbid†	Jordan	98	-66±3	-11±2	2.47±0.01
Europe	Apr	Rabat	Morocco	94	-60±3	-10±2	4.13±0.02
Europe	Apr	Fes	Morocco	94	-52±3	-13±2	0.83±0.02
Europe	Apr	Casablanca	Morocco	94	-49±3	-10±3	2.95±0.01
Europe	Apr	The Hague	Netherlands	80	-22.3±0.3	0.3±0.7	-3.08±0.06
Europe	Apr	Rotterdam	Netherlands	80	-13.7±0.1	0±1	-3.26±0.02
Europe	Apr	Amsterdam	Netherlands	80	2.8±0.7	0.3±0.6	-4.03±0.07
Europe	Apr	Warsaw	Poland	83	-7±4	-0±1	-3.951±0.008
Europe	Apr	Bucharest	Romania	87	-26±2	-20±2	0.18±0.03
Europe	Apr	Yekaterinburg	Russia	85	-81.5±0.9	25±1	-0.91±0.02
Europe	Apr	Chelyabinsk	Russia	85	-80.0±0.8	25±2	-2.465±0.006
Europe	Apr	Samara	Russia	85	-77±1	-45.3±0.7	-0.90±0.01
Europe	Apr	Ufa	Russia	85	-76±1	-13.9±0.9	-0.412±0.003
Europe	Apr	Kazan	Russia	85	-61±3	-55.5±0.3	-7.043±0.005
Europe	Apr	Saint Petersburg	Russia	85	-53±1	5.54±0.02	-0.07±0.08
Europe	Apr	Moscow	Russia	85	-44.0±0.2	-21.0±0.2	-2.19±0.02
Europe	Apr	Voronezh	Russia	85	-18±4	15±1	0.685±0.009
Europe	Apr	Rostov	Russia	85	-15±4	14.6±0.9	-0.13±0.02
Europe	Apr	Nizhniy Novgorod	Russia	85	22±5	27±2	-4.520±0.009
Europe	Apr	Belgrade	Serbia	98	-47±2	-10.8±0.8	2.28±0.05
Europe	Apr	Barcelona	Spain	85	-76±2	9.1±0.9	-4.9±0.1
Europe	Apr	Sevilla	Spain	85	-70±1	-6.6±1.0	-1.18±0.04
Europe	Apr	Madrid	Spain	85	-65±2	-3.2±0.2	-4.0±0.1
Europe	Apr	Damascus†	Syria	84	-33±3	-12±2	-2.10±0.02
Europe	Apr	Aleppo†	Syria	84	-28±2	-2±2	-5.18±0.02
Europe	Apr	Tunis†	Tunisia	91	-57±2	-5.2±0.9	0.81±0.01
Europe	Apr	Bursa	Turkey	76	-60±2	2±1	2.5±0.1
Europe	Apr	Antalya	Turkey	76	-58±2	5±2	8.05±0.03

Region	Month with strictest lockdown	City	Country	SI	Observed Change (%)	Expected change from meteorology (%)	Long-term trend (%)
Europe	Apr	Sanliurfa	Turkey	76	-55±2	7±3	7.07±0.01
Europe	Apr	Konya	Turkey	76	-52±2	8±1	5.25±0.02
Europe	Apr	Ankara	Turkey	76	-48±2	4.2±0.2	2.5±0.1
Europe	Apr	Gaziantep	Turkey	76	-47±2	1±2	8.03±0.02
Europe	Apr	Mersin	Turkey	76	-45±2	1±3	4.73±0.01
Europe	Apr	Izmir	Turkey	76	-31±2	6±1	3.97±0.07
Europe	Apr	Adana	Turkey	76	-29±3	3±2	3.35±0.01
Europe	Apr	Istanbul	Turkey	76	-27±3	13±2	1.98±0.10
Europe	Apr	Ashgabat[†]	Turkmenistan	39	-10±3	9±2	2.07±0.03
Europe	Apr	Kyiv[†]	Ukraine	89	-48±2	-25.5±0.7	2.68±0.02
Europe	Apr	Kharkiv[†]	Ukraine	89	-46±3	-13±1	-0.13±0.01
Europe	Apr	Odesa[†]	Ukraine	89	-41±2	-13±1	-1.12±0.03
Europe	Apr	Newcastle	United Kingdom	80	-67±2	-18±1	-2.45±0.02
Europe	Apr	London	United Kingdom	80	-51.2±0.1	-3.1±0.9	-3.68±0.02
Europe	Apr	Leeds	United Kingdom	80	-41±2	-9±1	-6.45±0.03
Europe	Apr	Birstall	United Kingdom	80	-41±2	-3.1±0.8	-4.71±0.04
Europe	Apr	Birmingham	United Kingdom	80	-33.9±0.8	-5.3±0.7	-4.90±0.07
Europe	Apr	Manchester	United Kingdom	80	-30±1	-8.6±0.7	-6.85±0.06
N. America	Apr	Toronto	Canada	73	-64±2	-50.9±0.6	-0.38±0.02
N. America	Apr	Calgary	Canada	73	-44±3	-1.0±0.9	1.09±0.02
N. America	Apr	Vancouver	Canada	73	10±31	-15.8±0.5	2.93±0.03
N. America	Apr	Edmonton	Canada	73	13±2	-7±2	-7.41±0.02
N. America	Apr	Montreal	Canada	73	18±4	-11±1	-5.50±0.04
N. America	Apr	San Pedro Sula[†]	Honduras	100	13±64	18±4	5.97±0.05
N. America	Apr	Puebla	Mexico	82	-31±4	4±4	4.90±0.06
N. America	Apr	Monterrey	Mexico	82	-25±9	-3.2±0.3	5.13±0.07
N. America	Apr	Juarez	Mexico	82	-24±3	-6±3	-3.12±0.03
N. America	Apr	Tijuana	Mexico	82	-20±3	-0±3	-3.15±0.03
N. America	Apr	Nezahualcoyotl	Mexico	82	-19±13	-12.4±0.1	0.3±0.1
N. America	Apr	Ecatepec	Mexico	82	-16±17	-12.4±0.1	1.8±0.1
N. America	Apr	Mexico City	Mexico	82	-16±31	-12.5±0.1	0.25±0.07
N. America	Apr	Leon de los Aldama	Mexico	82	-15±2	-3±3	5.51±0.06
N. America	Apr	Zapopan	Mexico	82	-15±8	-6±2	2.65±0.04
N. America	Apr	Guadalajara	Mexico	82	-12±12	-5.8±0.7	3.31±0.05
N. America	Apr	Atlanta	United States	79	-39±3	2.1±0.5	-7.80±0.01
N. America	Apr	Washington	United States	79	-38±3	-13.6±0.6	-4.07±0.02
N. America	Apr	Chicago	United States	79	-27.4±0.4	-23.9±0.8	-6.13±0.02
N. America	Apr	Philadelphia	United States	79	-21±1	-4±1	-5.71±0.01
N. America	Apr	New York	United States	79	-20.1±0.1	-4.1±1.0	-5.64±0.08
N. America	Apr	Los Angeles	United States	79	-12.2±0.8	7±1	-3.20±0.07

Region	Month with strictest lockdown	City	Country	SI	Observed Change (%)	Expected change from meteorology (%)	Long-term trend (%)
N. America	Apr	Boston	United States	79	-9±4	1±1	-3.26±0.02
N. America	Apr	Miami	United States	79	-7±6	17±5	-4.501±0.007
N. America	Apr	Dallas	United States	79	33±7	7±1	-3.24±0.03
N. America	Apr	Houston	United States	79	59±3	9±2	-2.39±0.02
N. America	May	Santo Domingo	Dominican Republic	92	-45±4	-9±3	2.63±0.06
N. America	May	Guatemala City†	Guatemala	95	-57±4	-1±3	-5.06±0.02
N. America	May	Valencia†	Venezuela	82	40±12	-2±6	-0.47±0.02
S. America	Apr	Buenos Aires†	Argentina	98	-41±3	15±2	-1.43±0.05
S. America	Apr	Guayaquil	Ecuador	94	-67±2	-2±2	-5.63±0.07
S. America	Apr	Lima	Peru	94	-72±2	-4±2	-0.03±0.01
S. America	Apr	Callao	Peru	94	-71.2±0.9	-4±5	2.665±0.008
S. America	Apr	Arequipa	Peru	94	-68±1	1±1	14.90±0.06
S. America	May	Rio de Janeiro	Brazil	75	-36±10	1.3±1.0	0.09±0.03
S. America	May	Sao Paulo	Brazil	75	-9±33	-14.4±0.6	-2.8±0.1
S. America	May	Belo Horizonte	Brazil	75	2±3	-6±1	-5.74±0.04
S. America	May	Campinas	Brazil	75	5.0±0.5	-6±2	1.240±0.008
S. America	May	Guarulhos	Brazil	75	11±20	-14±3	-1.99±0.08
S. America	May	Curitiba	Brazil	75	41±9	-33±3	-6.41±0.06
S. America	May	Porto Alegre	Brazil	75	45±5	-1±2	4.36±0.01
S. America	May	Caracas†	Venezuela	82	-28±4	-2±3	-11.41±0.06
S. America	Jun	Santiago	Chile	73	-22±75	-8.0±0.1	-0.10±0.01
Africa	Apr	Luanda†	Angola	88	-28±5	-1±3	-38.2±0.2
Africa	Apr	Shiraz	Iran	55	-18.3±0.6	-2±2	2.53±0.04
Africa	Apr	Ahvaz	Iran	55	-18±3	-1±3	-4.61±0.05
Africa	Apr	Esfahan	Iran	55	-2±16	5.2±0.7	0.0±0.1
Africa	Apr	Al Basrah	Iraq	93	-42±3	5±2	-9.16±0.02
Africa	Apr	Amman†	Jordan	98	-79±1	-11±2	-3.79±0.04
Africa	Apr	Port Harcourt†	Nigeria	84	-78±2	5±8	0.57±0.08
Africa	Apr	Lagos†	Nigeria	84	-66±3	-7±3	0.0±0.1
Africa	Apr	Onitsha†	Nigeria	84	-53±5	-9±3	0.17±0.03
Africa	Apr	Abuja†	Nigeria	84	-52±3	-5±2	3.49±0.05
Africa	Apr	Ibadan†	Nigeria	84	-38±5	-11±3	-2.05±0.10
Africa	Apr	Benin City†	Nigeria	84	-6±4	-8±5	-7.51±0.07
Africa	Apr	Doha†	Qatar	85	-32±8	-15±2	-8.3±0.1
Africa	Apr	Medina	Saudi Arabia	92	-52±2	7±2	-19.1±0.2
Africa	Apr	Mecca	Saudi Arabia	92	-51±2	8±2	-13.6±0.1
Africa	Apr	Jeddah	Saudi Arabia	92	-31±6	8±3	-21.5±0.2
Africa	Apr	Riyadh	Saudi Arabia	92	-27±8	-14.393±0.002	-13.1±1.0

Region	Month with strictest lockdown	City	Country	SI	Observed Change (%)	Expected change from meteorology (%)	Long-term trend (%)
Africa	Apr	Johannesburg†	South Africa	88	-50±4	-4±2	1.0±0.4
Africa	Apr	Vereeniging†	South Africa	88	-9±24	-4±2	-1.3±0.2
Africa	Apr	Sharjah†	United Arab Emirates	88	-13±3	-12±2	-0.2±0.3
Africa	Apr	Dubai†	United Arab Emirates	88	-9±5	-12±2	1.2±0.3
Africa	May	Alexandria†	Egypt	84	-32±3	-7±2	-0.90±0.10
Africa	May	Shubra al Khaymah†	Egypt	84	-17±6	-2±1	-7.22±0.07
Africa	May	Cairo†	Egypt	84	-12±15	-2.4±0.3	-4.7±0.2
Africa	May	Giza†	Egypt	84	-11±15	-2.5±0.2	-5.2±0.2
Africa	May	Tripoli†	Libya	95	0.6±0.5	-13±3	-0.95±0.02
Africa	Jun	Matola†	Mozambique	56	-39±10	-10±1	-2.2±0.3
Africa	Jun	Maputo†	Mozambique	56	-23±15	-10±2	-0.4±0.3
Oceania	Apr	Melbourne	Australia	71	-21±15	21.7±0.8	-2.42±0.02
Oceania	Apr	Brisbane	Australia	71	-0.3±0.9	36±2	-2.19±0.01
Oceania	Apr	Sydney	Australia	71	2±5	61±5	-1.30±0.01
Oceania	May	Patam†	Indonesia	68	-21±1	-8±3	-3.185±0.004
Average					-32±2	-1±1	-1.5±0.4

Supplemental Figures

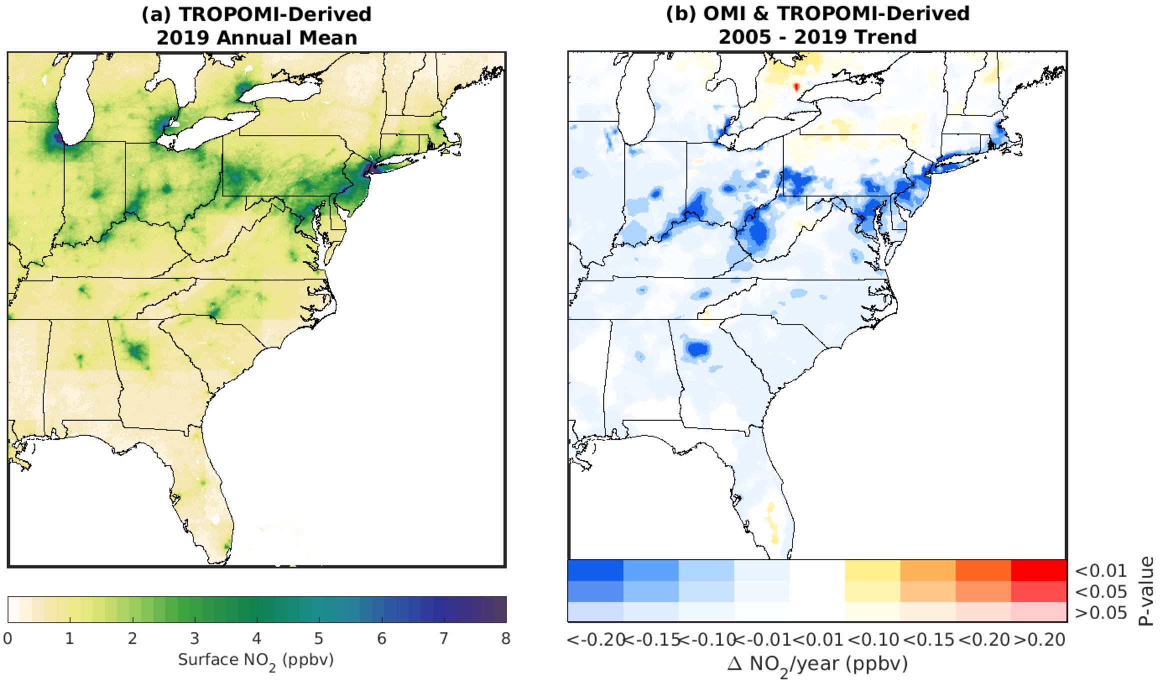

Supplemental Figure 1. (Left) Multi-year (2005-2019) satellite-derived surface NO_2 trends across eastern North America. (Right) 2019 annual mean TROPOMI-derived surface NO_2 concentrations.

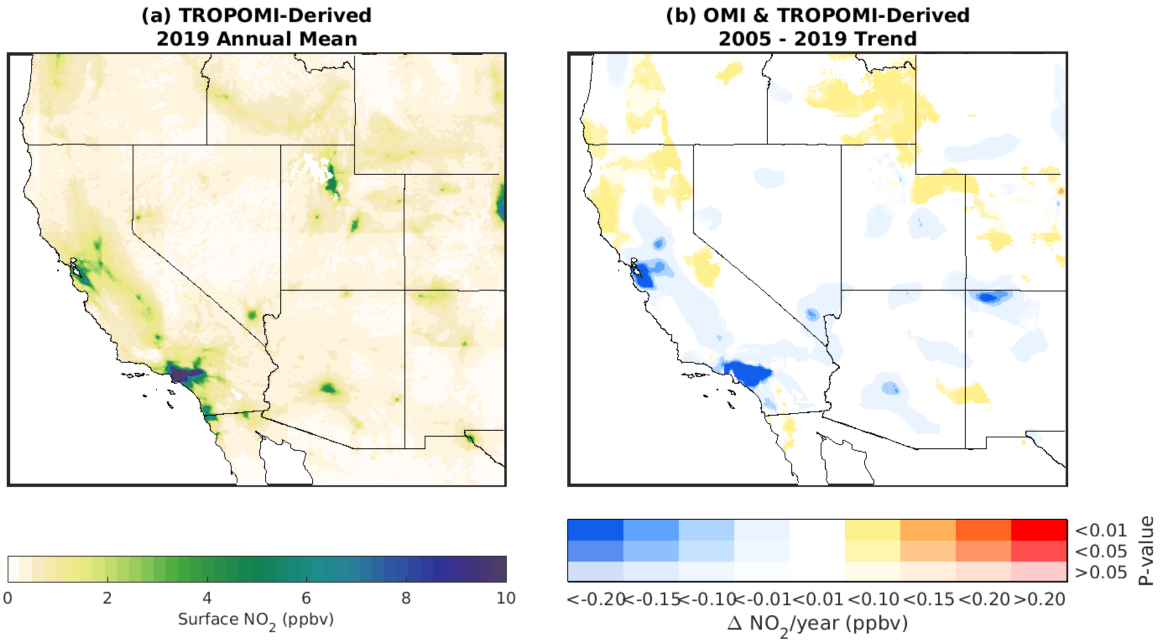

Supplemental Figure 2. (Left) Multi-year (2005-2019) satellite-derived surface NO_2 trends across western North America. (Right) 2019 annual mean TROPOMI-derived surface NO_2 concentrations.

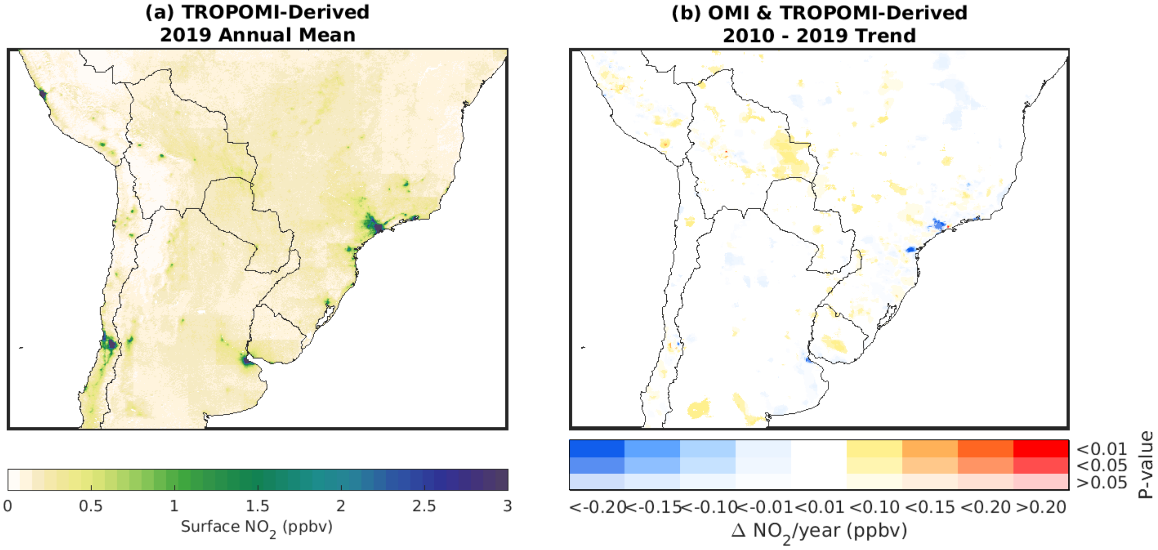

Supplemental Figure 3. (Left) Multi-year (2010-2019) satellite-derived surface NO_2 trends across central South America. (Right) 2019 annual mean TROPOMI-derived surface NO_2 concentrations.

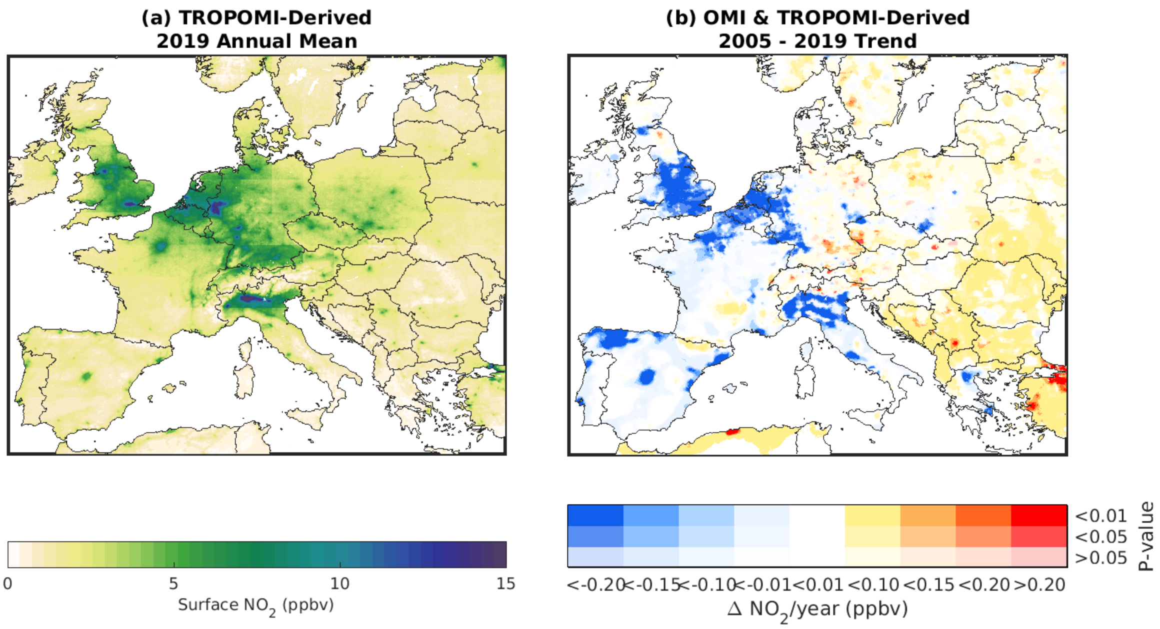

Supplemental Figure 4. (Left) Multi-year (2005-2019) satellite-derived surface NO_2 trends across Europe. (Right) 2019 annual mean TROPOMI-derived surface NO_2 concentrations.

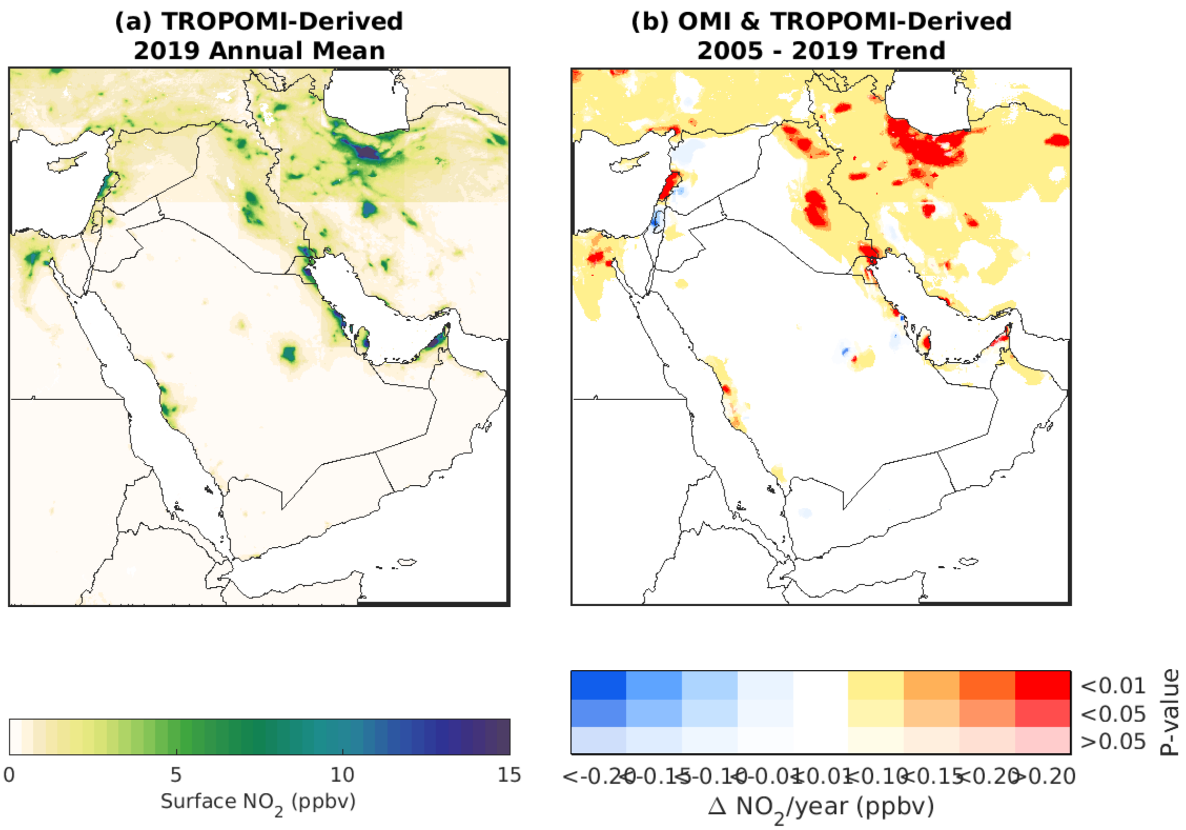

Supplemental Figure 5. (Left) Multi-year (2005-2019) satellite-derived surface NO_2 trends across the Middle East. (Right) 2019 annual mean TROPOMI-derived surface NO_2 concentrations.

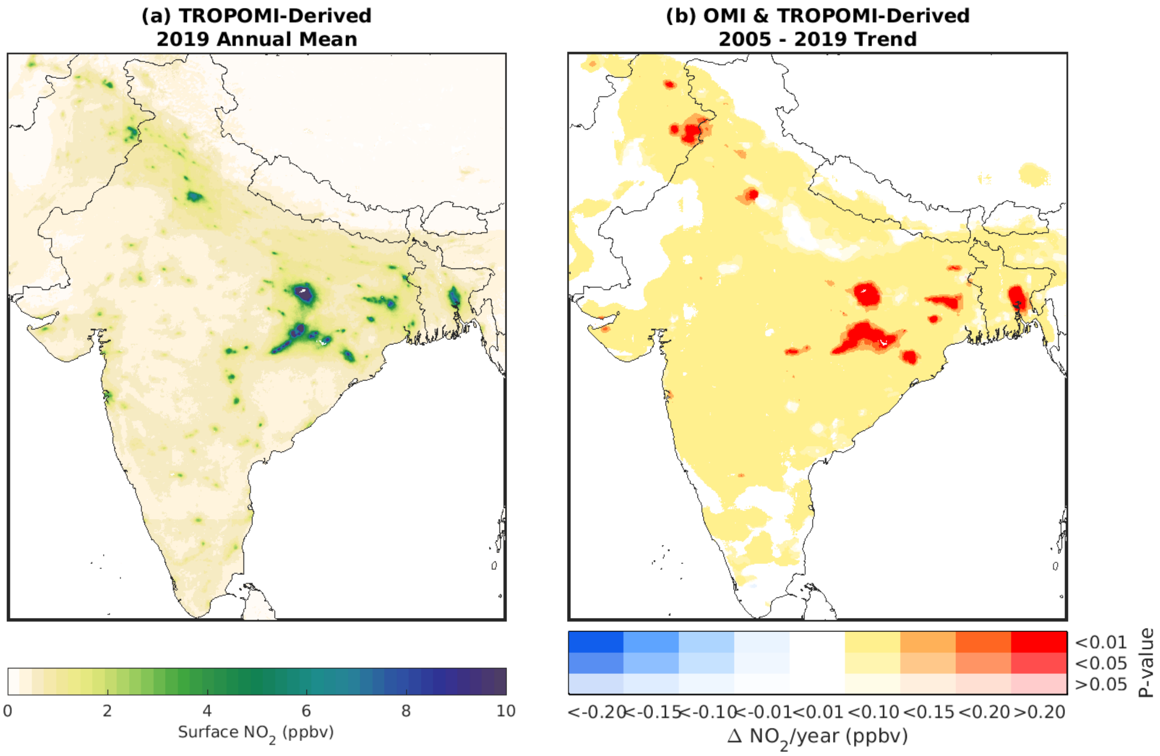

Supplemental Figure 6. (Left) Multi-year (2005-2019) satellite-derived surface NO_2 trends across India. (Right) 2019 annual mean TROPOMI-derived surface NO_2 concentrations.

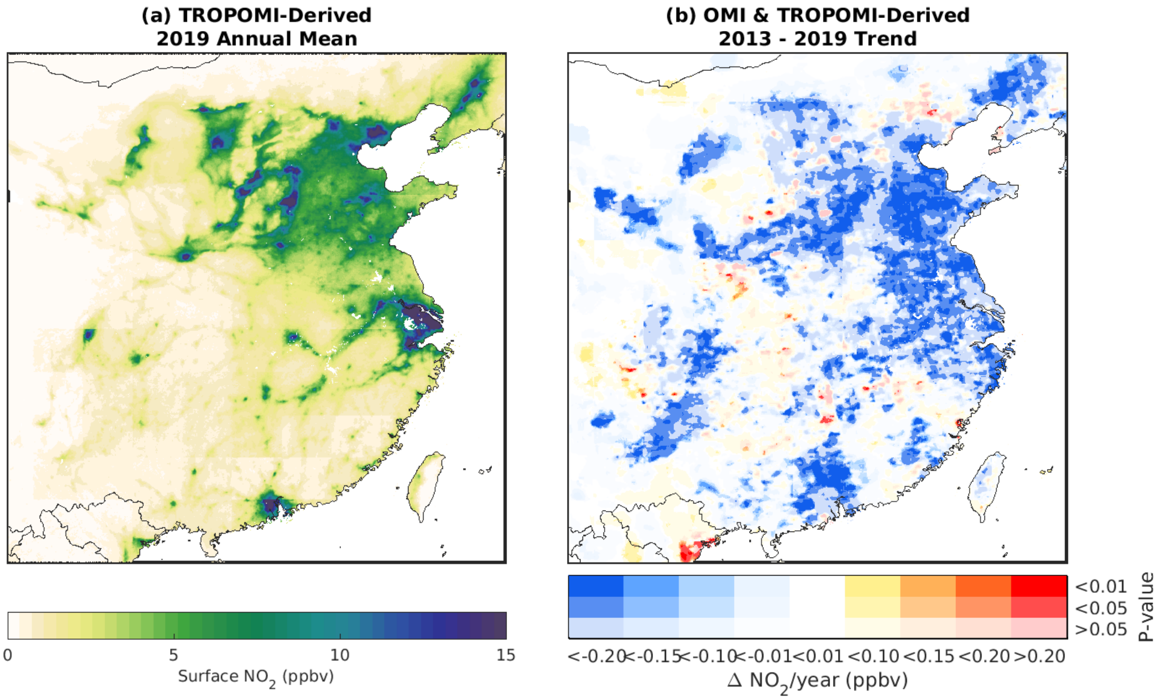

Supplemental Figure 7. (Left) Multi-year (2013-2019) satellite-derived surface NO_2 trends across China. (Right) 2019 annual mean TROPOMI-derived surface NO_2 concentrations.

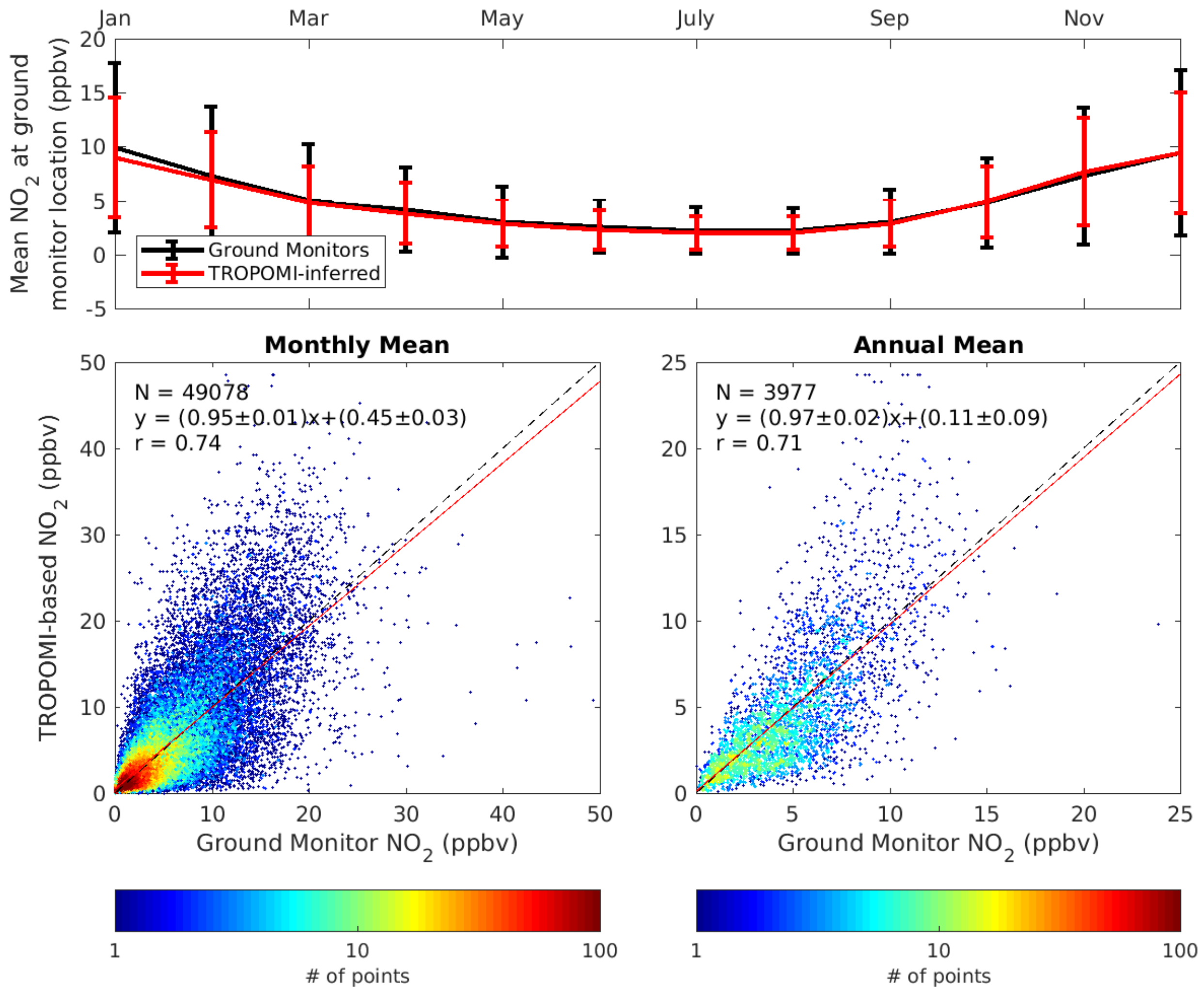

Supplement Figure 8: (Top) Global monthly mean NO_2 concentrations at ground monitor locations in 2019. Error bars indicate standard deviations. (Bottom) Comparison between monthly mean (left) and annual mean (right) TROPOMI-derived with ground monitor NO_2 observations. Each data point represents a monthly (left) or annual (right) value for an individual monitor location.

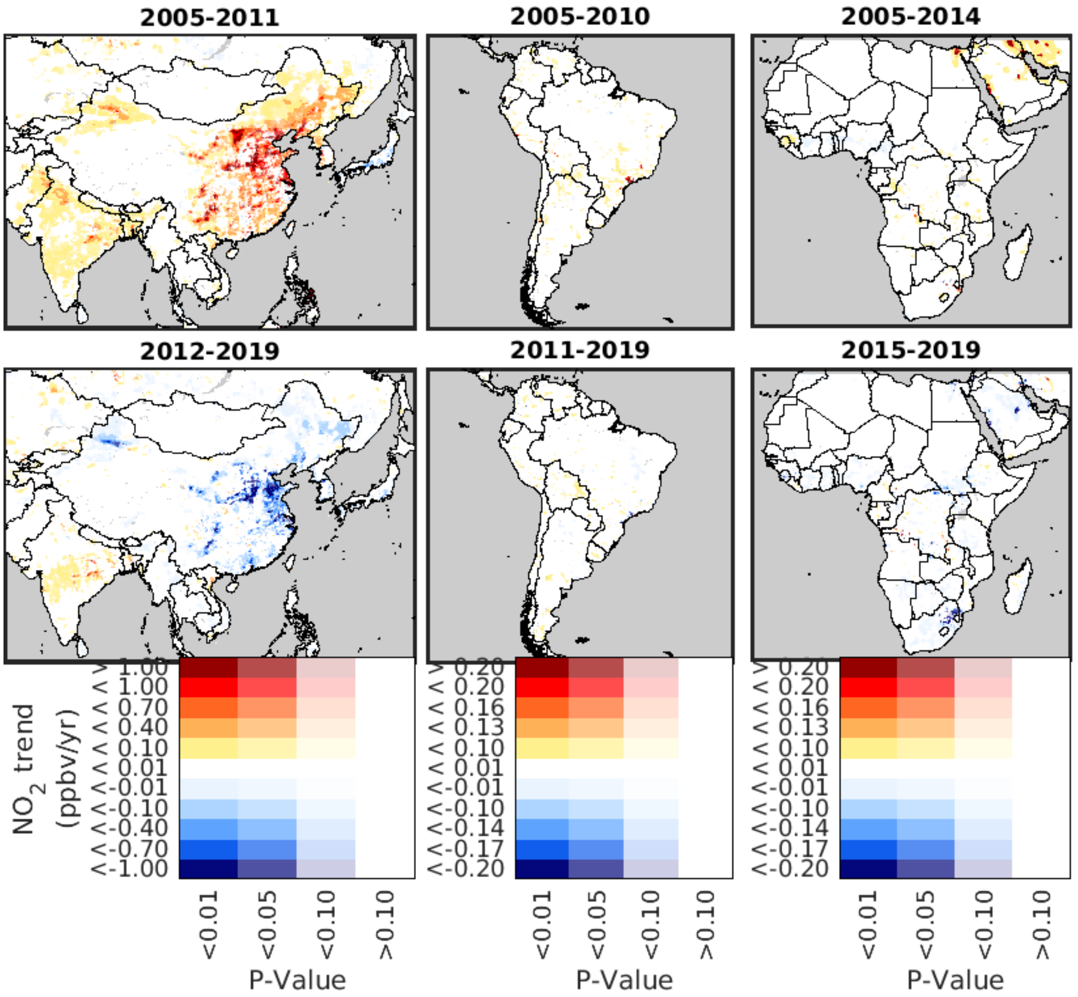

Supplemental Figure 9: Multi-year satellite-derived surface NO_2 trends across Asia, South America, and Africa. Color intensity represents statistical significance of trends. Ocean values are masked in grey.

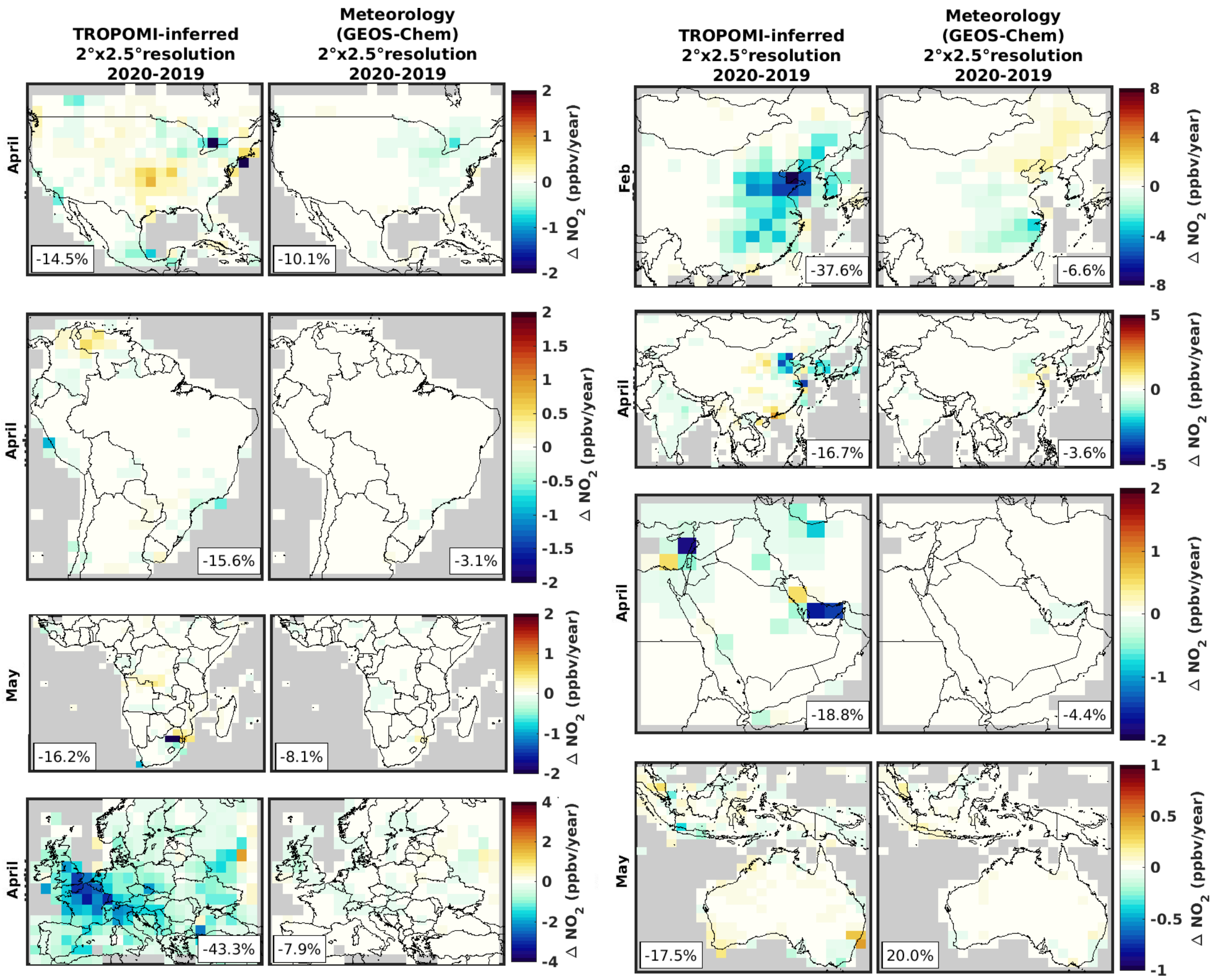

Supplemental Figure 10: (Top) TROPOMI-derived monthly mean NO_2 difference aggregated to 2°x2.5° resolution for comparison with simulated values. (Bottom) GEOS-Chem-simulated ground-level NO_2 difference. GEOS-Chem emissions are the same in 2020 and 2019, so all simulated 2020-2019 differences are from meteorological impacts on NO_2. Grey indicates ocean regions or areas with persistent cloud or snow cover.

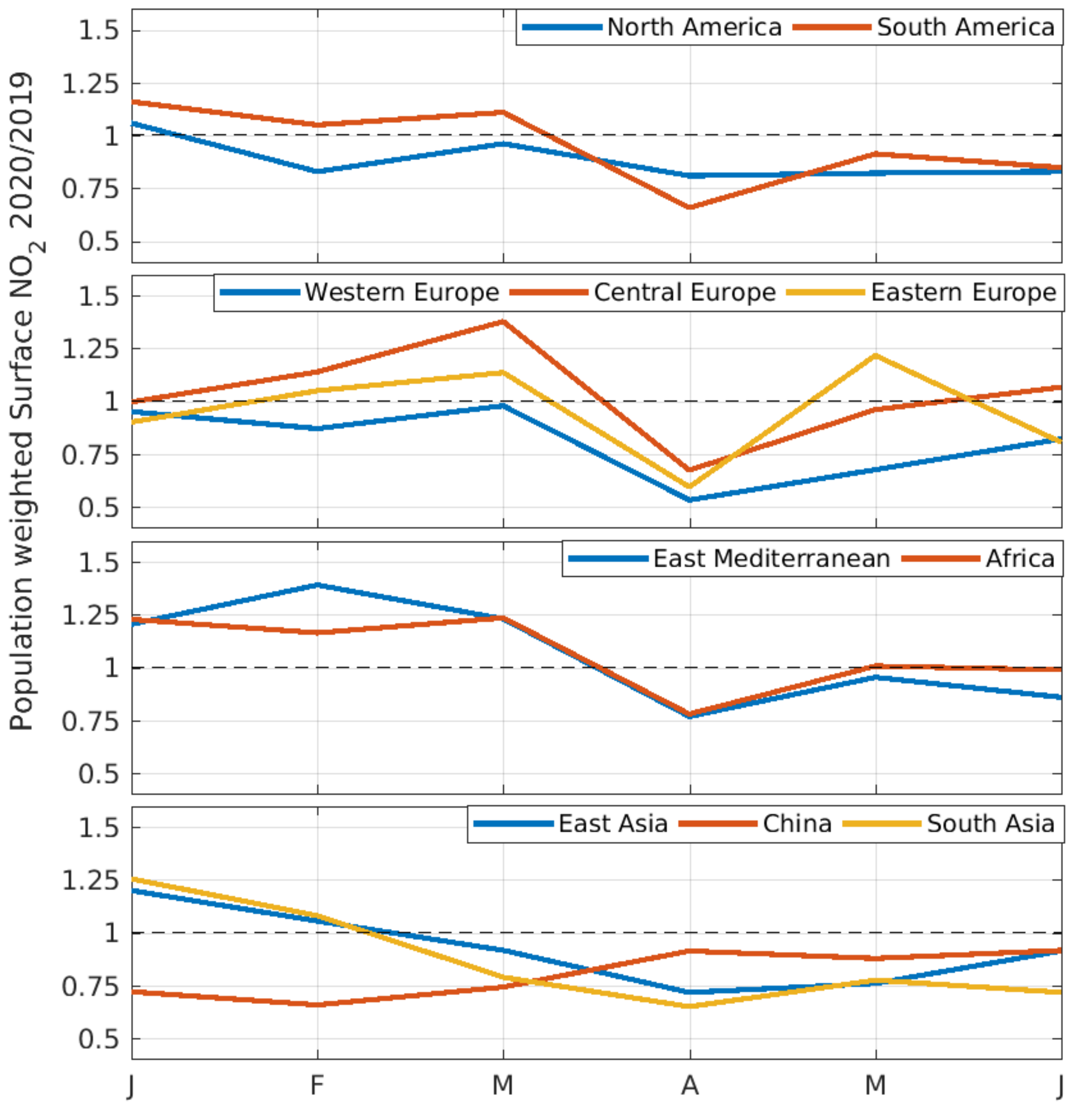

Supplemental Figure 11: Ratio of monthly population-weighted TROPOMI-derived surface NO_2 at 1x1 km^2 in 2020 to 2019.

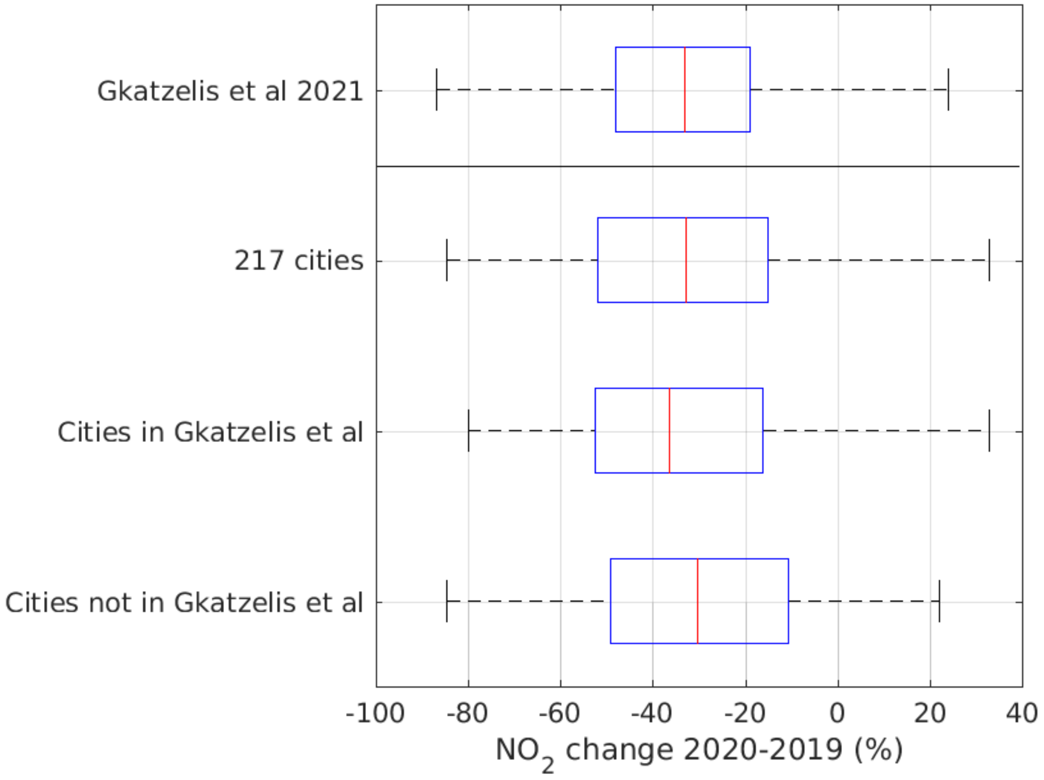

Supplemental Figure 12: Box plots of 2020-2019 NO_2 changes. From top: changes in ground monitor observations as summarized in Gkatzelis et al., TROPOMI-derived changes from 215 cities included in Supplemental Table 1, TROPOMI-derived changes from the 150 cities that are included in the Gkatzelis et al review, TROPOMI-derived changes from the 65 cities not included in the review paper. Red lines indicate median values, blue box indicates 25th-75th percentile values, and dashed lines indicate the data range.

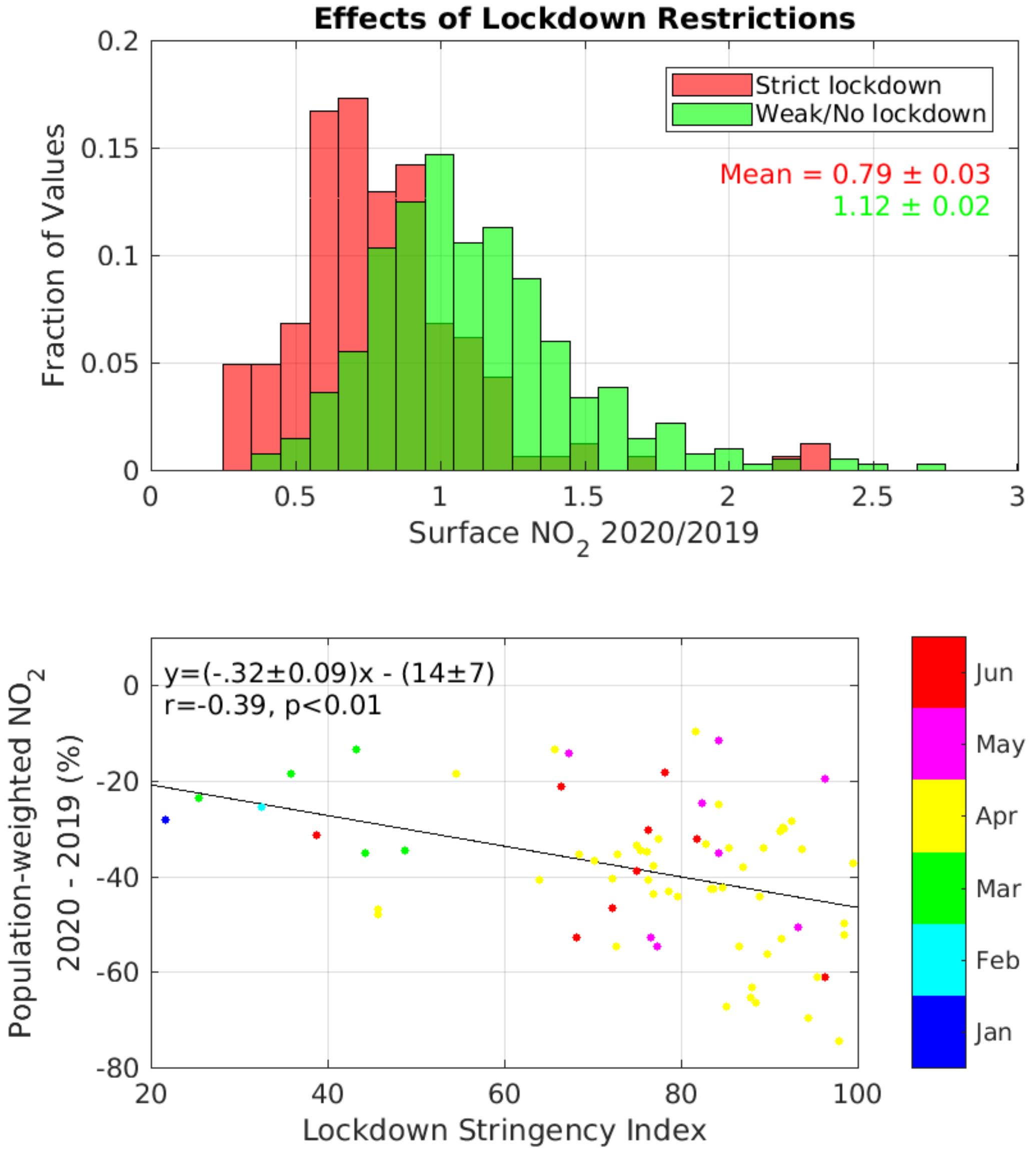

Supplemental Figure 13: (Top) Ratio of TROPOMI-derived population-weighted monthly mean 2020/2019 ratio as a function of lockdown stringency for all countries worldwide. Lockdown stringency is defined using Oxford COVID-19 Government Response Tracker Stringency Index where "strict lockdown" are countries and months where the monthly minimum Stringency Index is above the 75% percentile, and "weak/no lockdown" where monthly median Stringency Index is below the 25% percentile. Mean values inset. Darker green colour indicates area of overlap between the distributions. (Bottom) Percent change in monthly mean population-weighted NO_2 for all countries for the month with the strictest lockdown stringency.

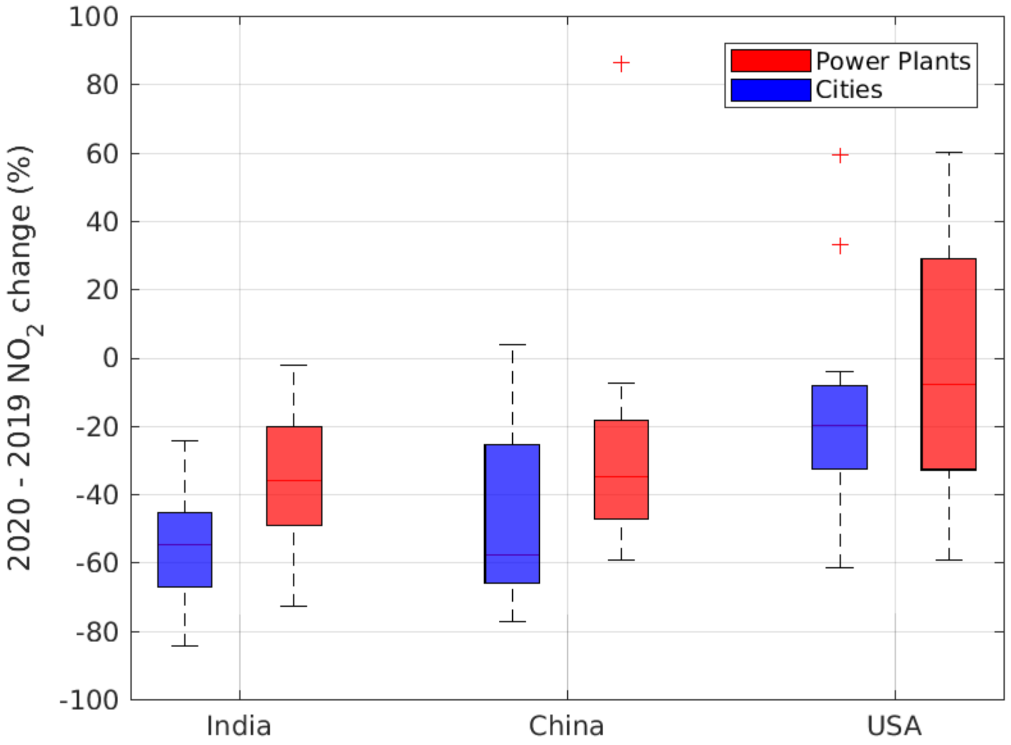

Supplemental Figure 14: Comparison between 2020-2019 monthly mean NO_2 concentrations at 20 most populous cities (blue) and 20 largest coal, oil, or gas-fueled power generation facilities (red). Concentrations are calculated over a 20x20 km^2 area surrounding the city or facility. February values are used in China, while April is used in India and the US to focus on the month with the largest change. Lines mid-box indicate median values, box boundaries indicate 25th-75th percentile values, and dashed lines indicate the data range with outliers marked by red +.

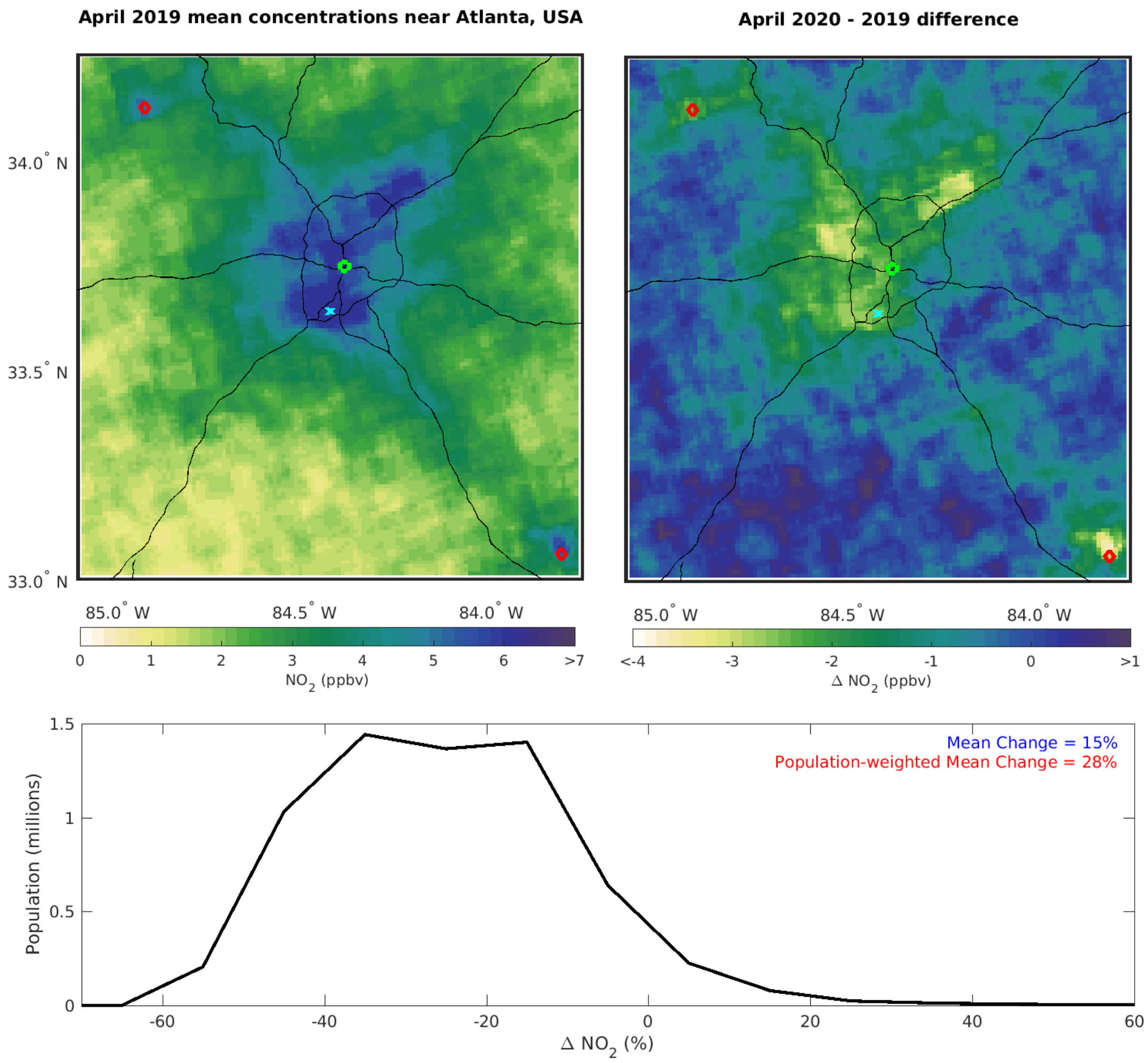

Supplemental Figure 15: (Top) April 2019 and 2020-2019 difference of TROPOMI-inferred ground level NO_2 mixing ratio near Atlanta, USA. Green circle represents downtown Atlanta, red diamonds represent coal-burning power plants with capacities > 2000 MW. Blue *x* represents Hartsfield-Jackson International Airport. Black lines indicate major highways. (Bottom) Change in population NO_2 exposure.

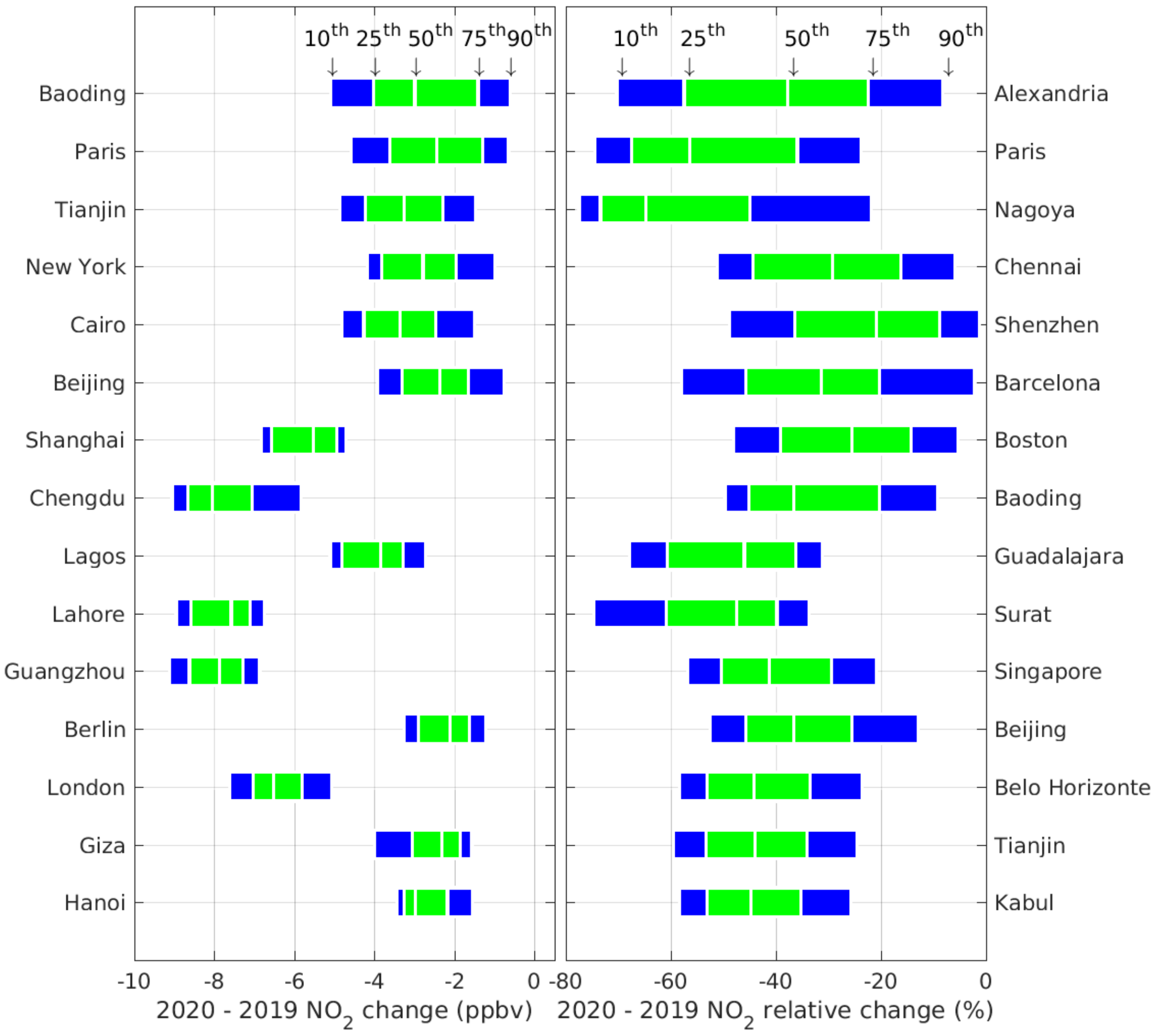

Supplemental Figure 16: Distributions of absolute (left) and relative (right) changes in population NO_2 exposure during lockdowns, for the 12 cities with populations greater than 3 million with the greatest range of exposures. Bars represent percentiles of the city's population that experienced changes of the given magnitude (i.e. Leftmost edge of bars represents the concentration change exceeded by 10% of the city's population, middle of green bar represents the median change in population NO2 exposure, rightmost edge is the 90th percentile of population exposure).

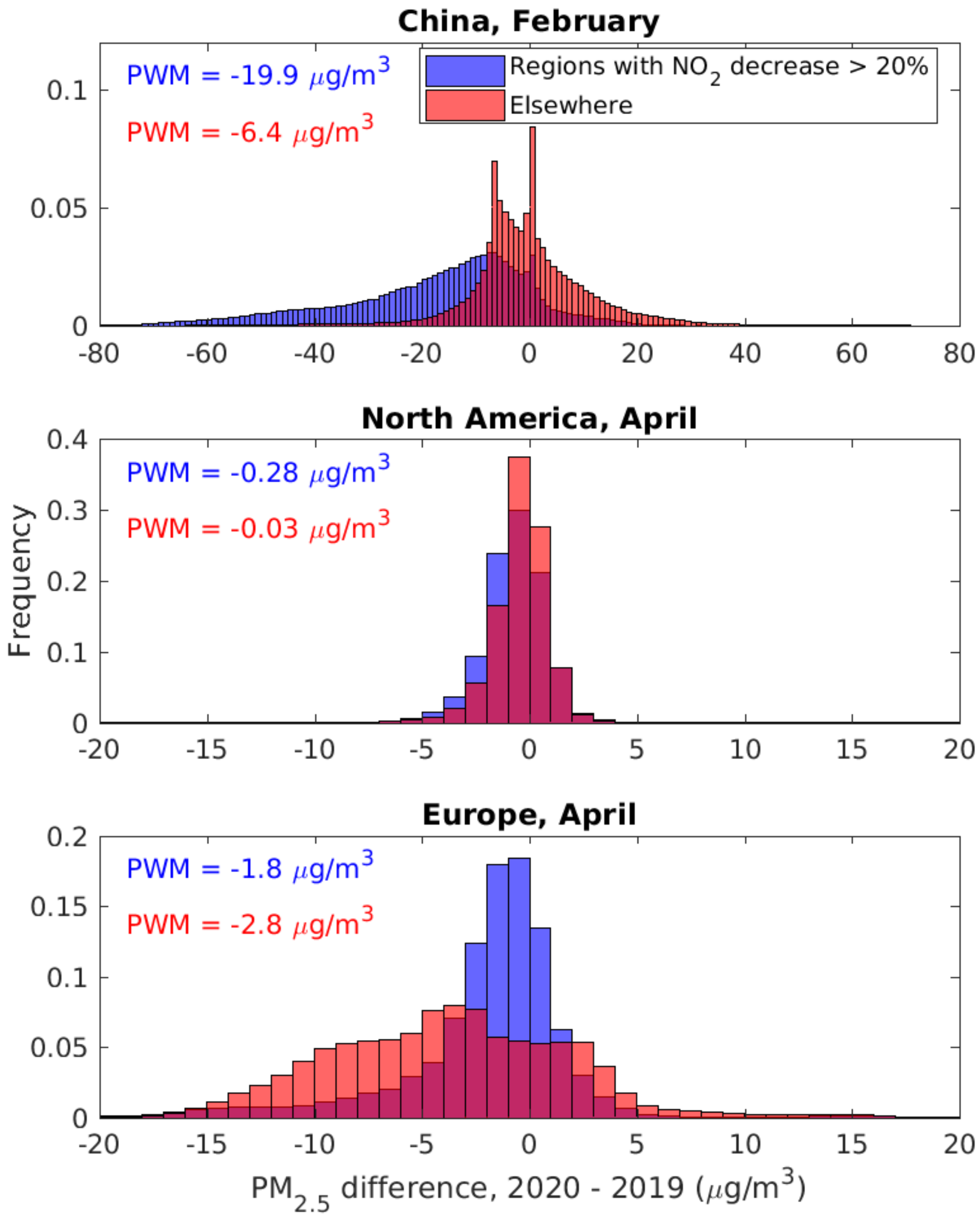

Supplemental Figure 17: $PM_{2.5}$ concentration changes from 2020-2019. Distributions describe change in monthly mean satellite-derived $PM_{2.5}$ concentrations gridded at ~1x1 km^2 resolution for February in China and April in Europe and North America. Blue distributions represent grid boxes where 2020-2019 monthly mean TROPOMI-derived ground level NO_2 concentration decreases exceed the 90th percentile. Red distributions describe all other grid boxes. Population-weighted mean $PM_{2.5}$ concentration changes are inset.

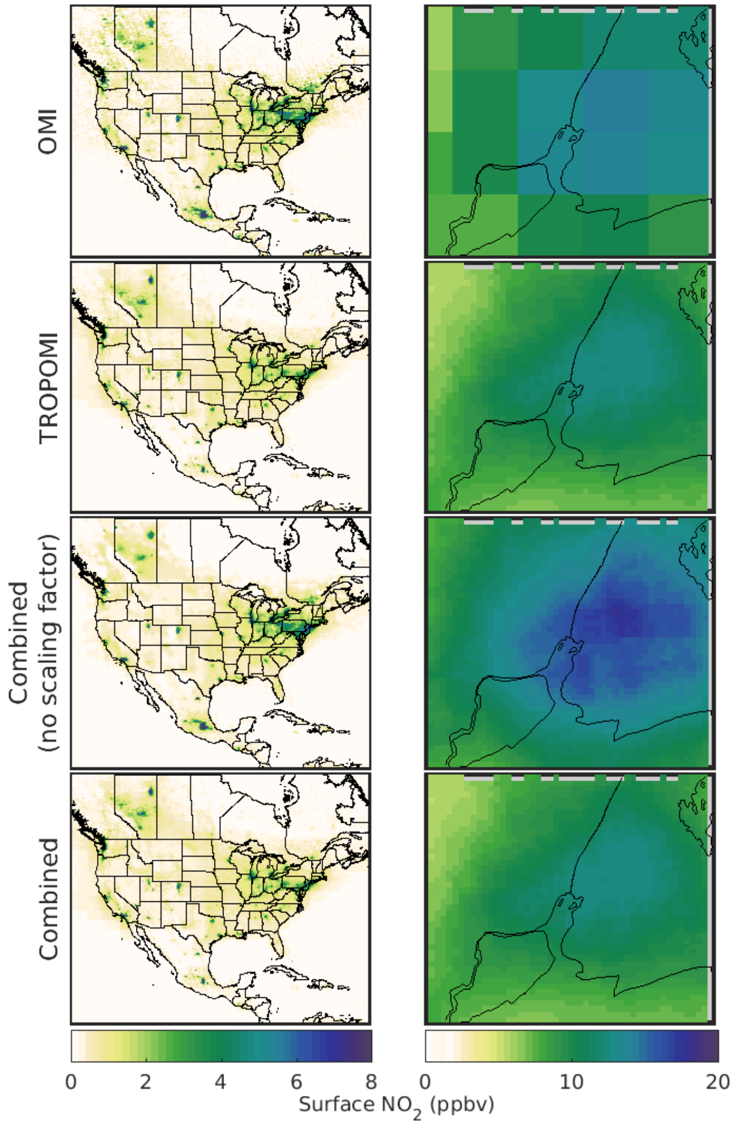

Supplement Figure 18: Annual mean surface NO_2 for 2019 for North America (left) and New York City (right). From top: derived from OMI NO_2 columns at ~10x10 km^2, TROPOMI columns at ~1x1 km^2, OMI and TROPOMI combined NO_2 columns at ~1x1 km^2 without applying the TROPOMI/OMI surface ratio scaling factor, and combined NO_2 columns at ~1x1 km^2 with the scaling factor.

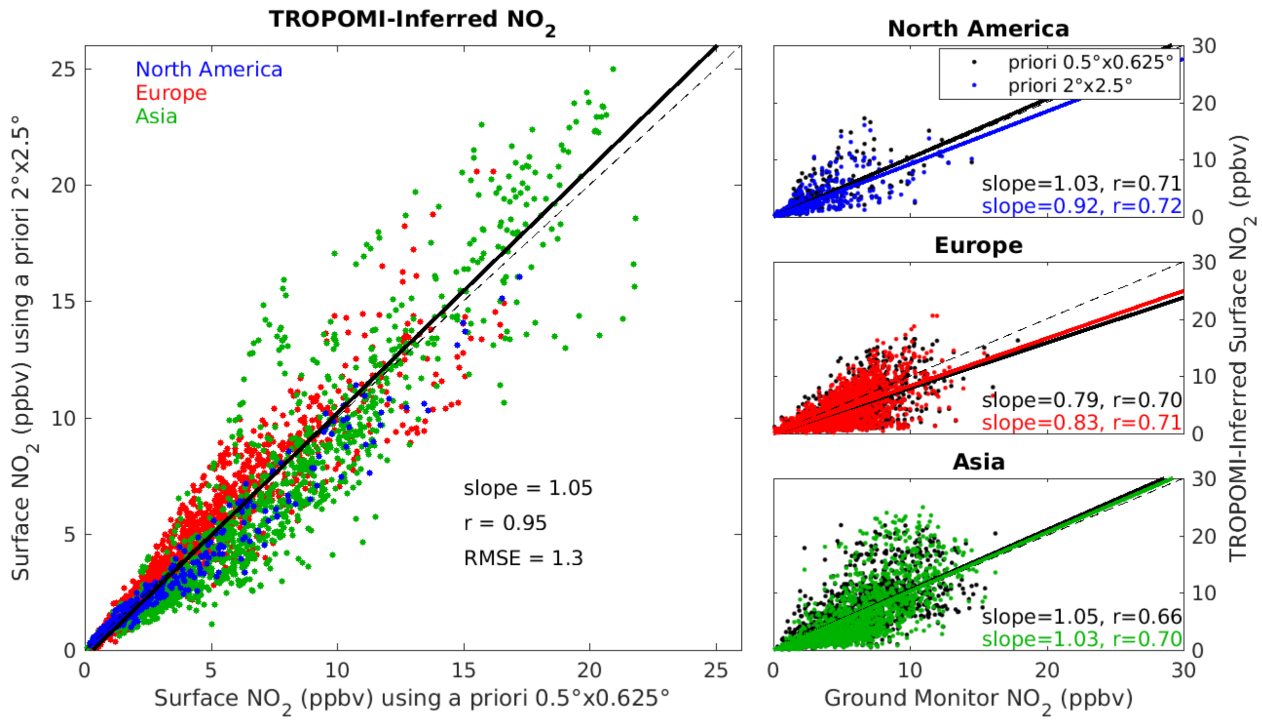

Supplemental Figure 19: Comparison of TROPOMI-derived 2019 annual mean surface concentrations across North America, Europe, and Asia, calculated using simulated information at either 2°x2.5° or 0.5°x0.625° resolution.

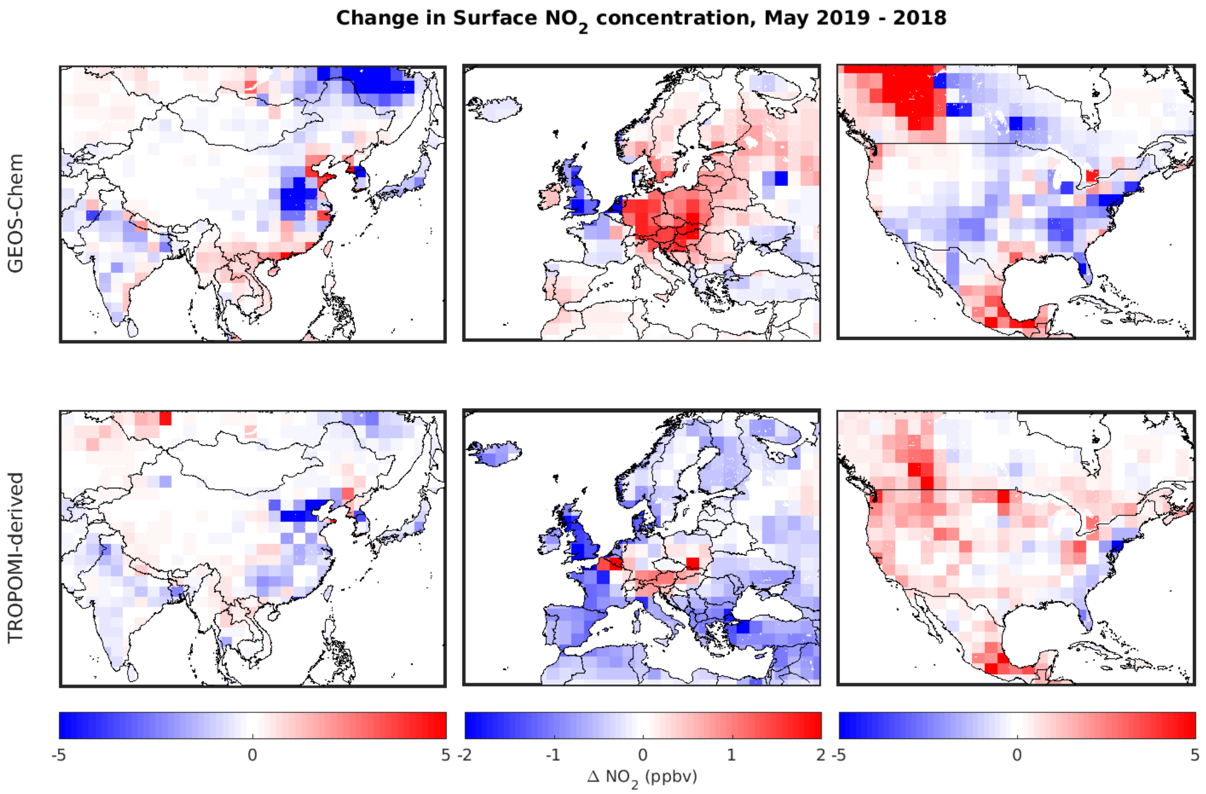

Supplemental Figure 20: Comparison of changes in TROPOMI-derived ground-level concentrations for May 2018-2019 with GEOS-Chem-simulated changes. May 2018-2019 is chosen for this example as it is a similar season as the lockdown period examined here and TROPOMI observations in April 2018 are not available. Direct comparisons of TROPOMI-derived changes and simulated changes will reflect differences resulting from the higher resolution of TROPOMI and potential biases in emission inventories, however GEOS-Chem generally reproduces most of the observed features (increases in southeast Asia, central Europe, western Canada, and Mexico; decreases in eastern China, Korea, Japan, India, UK, eastern US) lending confidence that GEOS-Chem can represent meteorologically-driven changes in NO_2.

References:

1. Cooper, M. J., Martin, R. V, McLinden, C. A. & Brook, J. R. Inferring ground-level nitrogen dioxide concentrations at fine spatial resolution applied to the TROPOMI satellite instrument. *Environ. Res. Lett.* **15**, 104013 (2020).

2. Gkatzelis, G. I. *et al.* The global impacts of COVID-19 lockdowns on urban air pollution: A critical review and recommendations. *Elem. Sci. Anthr.* **9**, (2021).
